# Supplementary material for: Single-exonuclease nanocircuits reveal the RNA degradation dynamics of PNPase and demonstrate potential for RNA sequencing
Source: Nat Commun. 2023 Feb 1;14:552. doi: 10.1038/s41467-023-36278-6 (PMC9892577; doi:10.1038/s41467-023-36278-6)
Supplement: Supplementary file 1 — Supplementary Information [file 41467_2023_36278_MOESM1_ESM.pdf]

Supplementary Information for

Single-exonuclease nanocircuits reveal the RNA degradation dynamics of PNPase and demonstrate potential for RNA sequencing

Zhiheng Yang[1,2], Wenzhe Liu[2], Lihua Zhao[2], Dongbao Yin[1,2], Jianfei Feng[2], Lidong Li[1,]* & Xuefeng Guo[2,3]*

[1]State Key Laboratory for Advanced Metals and Materials, School of Materials Science and Engineering, University of Science and Technology Beijing, Beijing 100083, P. R. China.

[2]Beijing National Laboratory for Molecular Sciences, National Biomedical Imaging Centre, College of Chemistry and Molecular Engineering, Peking University, 292 Chengfu Road, Haidian District, Beijing 100871, P. R. China.

[3]Centre of Single-Molecule Sciences, Institute of Modern Optics, Frontiers Science Centre for New Organic Matter, Tianjin Key Laboratory of Micro-scale Optical Information Science and Technology, College of Electronic Information and Optical Engineering, Nankai University, 38 Tongyan Road, Jinnan District, Tianjin 300350, P. R. China.

*Corresponding authors. Email: guoxf@pku.edu.cn (X.G.); lidong@mater.ustb.edu.cn (L.L.)

Table of Contents

Supplementary Note 1. The device fabrication process.

Supplementary Note 2. Electrical characterization of SiNW FETs.

Supplementary Note 3. The immobilization process of a single *E. coli* PNPase protein.

Supplementary Note 4. The complete sequence of *E. coli* PNPase core regions and location information of the mutation site.

Supplementary Note 5. Systematic analysis of statistical data from the RNA analog binding process.

Supplementary Note 6. Systematic analysis of statistical data from the RNA analog degradation process.

Supplementary Note 7. Statistical analysis of fingerprint data from the degradation process of the heterogeneous sequence.

Supplementary Note 8. The assay data of the regulation on the Debye length in the buffer solution.

Supplementary Note 1. The device fabrication process.

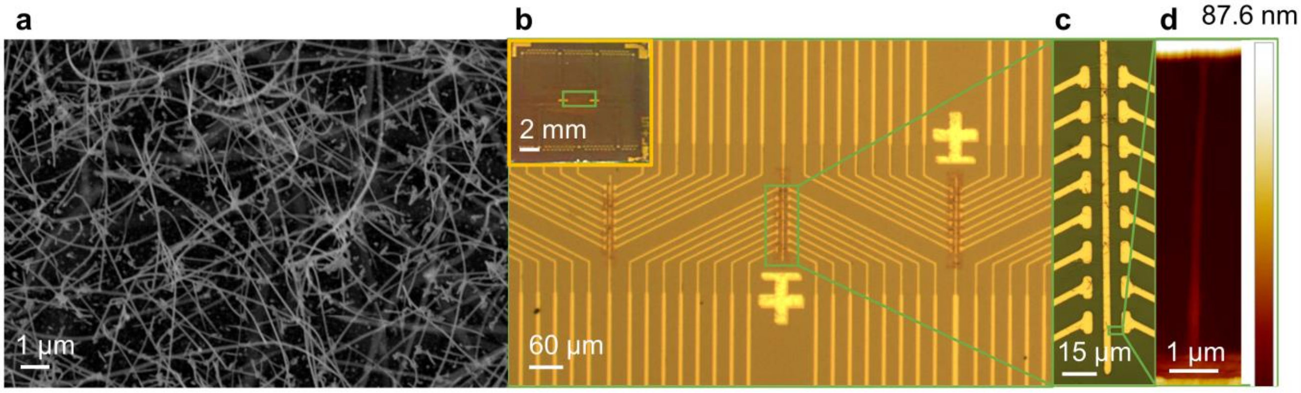

Supplementary Fig. 1 | SEM and optical images of a high–density SiNW FET array. (a) SEM image of SiNWs grown by gold-catalyzed CVD. Experiments were repeatedly conducted three times with similar results and one representative image was chosen for analysis. (b) optical images of SiNW-FET arrays. The inset shows a pattern we designed with 96 pairs of electrodes to make transistor arrays. Experiments were repeatedly conducted three times with similar results and one representative image was chosen for analysis. (c) magnified optical image of electrode couples. (d) AFM image of a single silicon nanowire between electrode couples. Experiments were repeatedly conducted three times with similar results and one representative image was chosen for analysis.

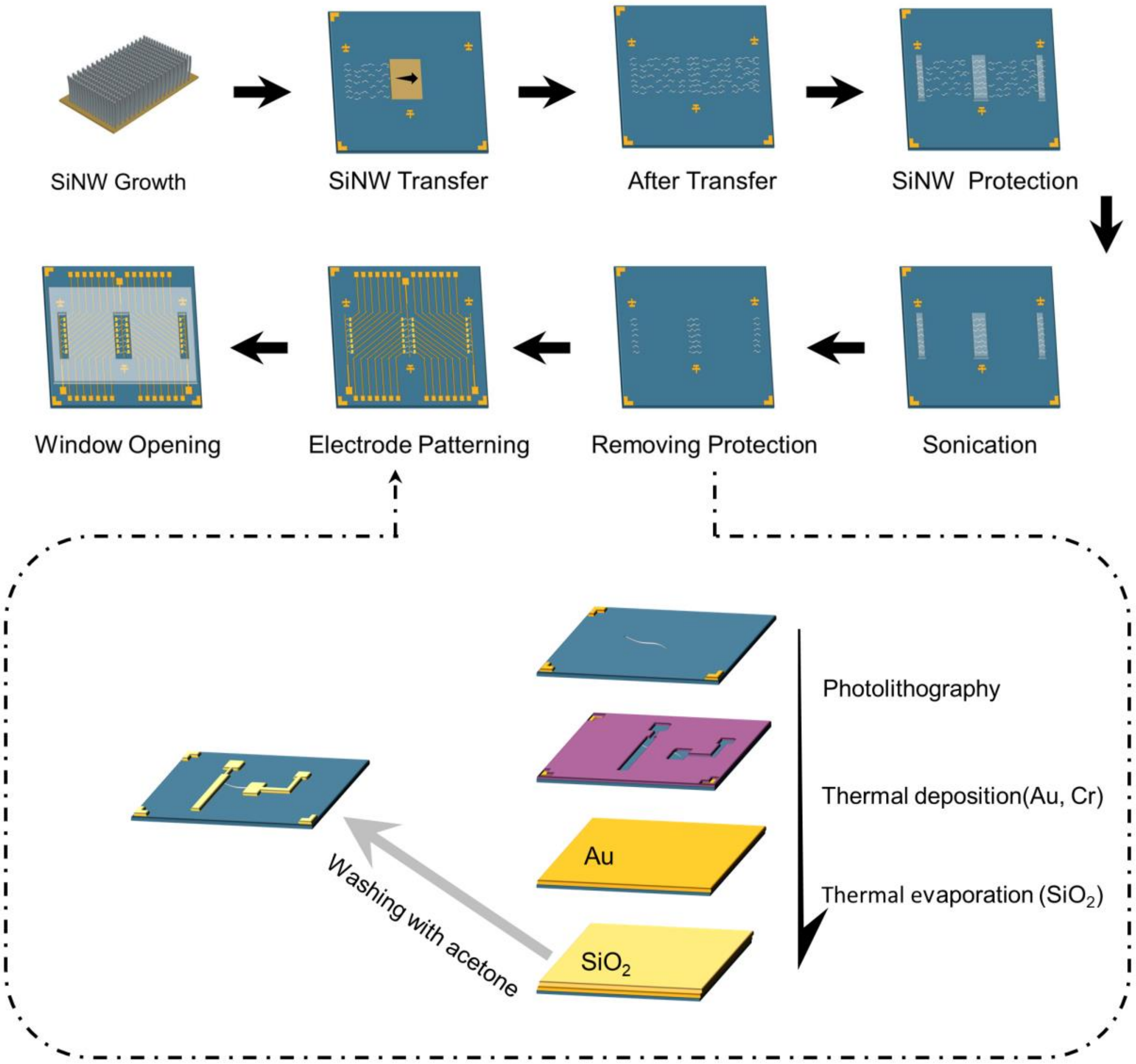

Supplementary Fig. 2 | The brief process of mechano–sliding transfer of SiNWs and FET array fabrication. The complete electrode patterning process was illustrated inside the dotted box.

Supplementary Note 2. Electrical characterization of SiNW FETs.

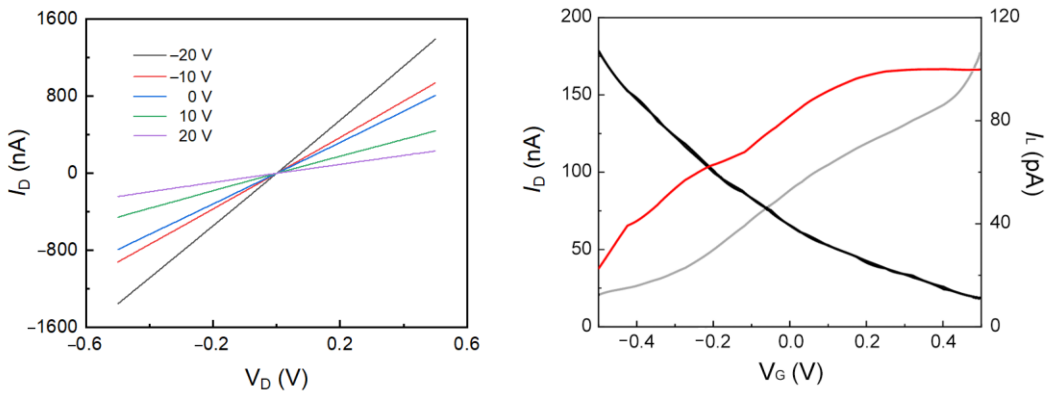

Supplementary Fig. 3 | Output characteristics for a *p*–type SiNW FET device. Left: I–V_D curves of the SiNW at different gate voltages (–20, –10, 0, 10, and 20 V) when V_D scans from 0.5 V to –0.5 V. Right: Source/drain current curves (one circle) of the SiNW in the buffer solution (black curve) and the corresponding leakage currents of the device in the buffer solution (red curve: from –0.5 V to 0.5 V and grey curve: from 0.5 V to –0.5 V). The probe of the gate voltage was immersed into the buffer solution from the top.

Supplementary Note 3. The immobilization process of a single *E. coli* PNPase protein.

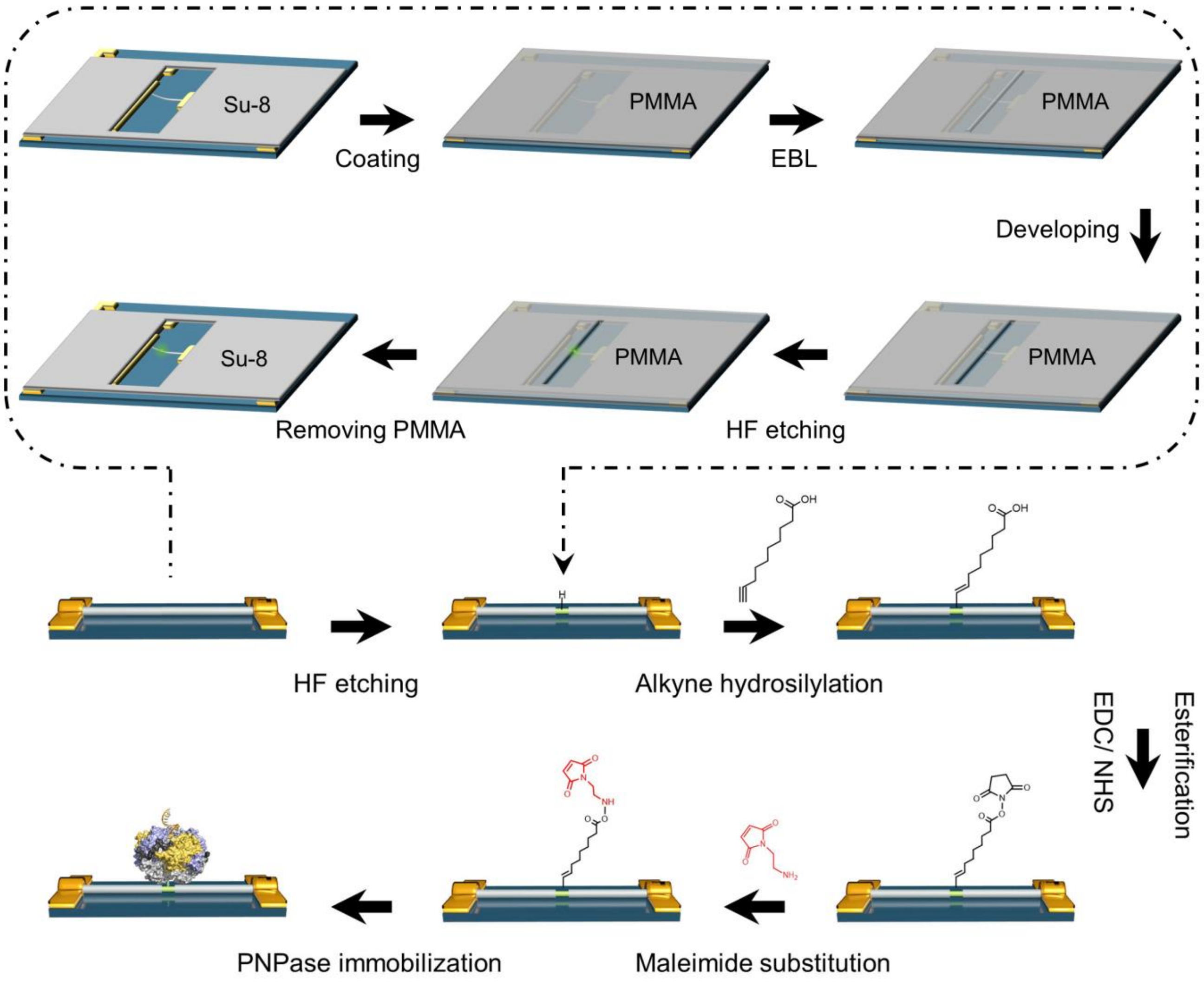

Supplementary Fig. 4 | Schematic demonstration of the strategy used for surface functionalization and single PNPase protein immobilization. The generation routine of the special area for the confinement conjunction was illustrated inside the dotted box.

Supplementary Note 4. The complete sequence of *E. coli* PNPase core regions and location information of the mutation site.

Supplementary Table 1 | Complete sequences of the *E. coli* PNPase core region

Before mutation

1 mlnpivrkfq ygqhtvtlet gmmarqataa vmvsmddtav fvtvvgqkka kpgqdffplt
61 vnyqertyaa gripgsffrr egrpsegetl iarlidrpir plfpegfvne vqviatvvsv
121 npqvnpdiva migasaalsl sgipfngpig aarvgyindq yvlnptqdel keskldlvva
181 gteaavlmve seaqllsedq mlgavvfghe qqqvviqnin elvkeagkpr wdwqpepvne
241 alnarvaala earlsdayri tdkqeryaqv dviksetiat llaedetlde nelgeilhai
301 eknvvrsrvl agepridgre kdmirgldvr tgvlprthgs alftrgetqa lvtatlgtar
361 daqvldelmg ertdtflfhy nfppysvget gmvgspkrre ighgrlakrg vlavmpdmdk
421 fpytvrvvse itesngsssm asvcgaslal mdagvpikaa vagiamglvk egdnyvvlsd
481 ilgdedhlgd mdfkvagsrd gisalqmdik iegitkeimq valnqakgar lhilgvmeqa
541 inaprgdise faprihtiki npdkikdvig kggsviralt eetgttieie ddgtvkiaat
601 dgekakhair rieeitaeie vgrvytgkvt rivdfgafva iggg keglvh isqiadkrve
661 kvtdylqmgq evpvkvlevd rqgrirlsik eateqsqpaa apeapaaeqg e

After mutation

1 mlnpivrkfq ygqhtvtlet gmmarqataa vmvsmddtav fvtvvgqkka kpgqdffplt
61 vnyqertyaa gripgsffrr egrpsegetl iarlidrpir plfpegfvne vqviatvvsv
121 npqvnpdiva migasaalsl sgipfngpig aarvgyindq yvlnptqdel keskldlvva
181 gteaavlmve seaqllsedq mlgavvfghe qqqvviqnin elvkeagkpr wdwqpepvne
241 alnarvaala earlsdayri tdkqeryaqv dviksetiat llcedetlde nelgeilhai
301 eknvvrsrvl agepridgre kdmirgldvr tgvlprthgs alftrgetqa lvtatlgtar
361 daqvldelmg ertdtflfhy nfppysvget gmvgspkrre ighgrlakrg vlavmpdmdk
421 fpytvrvvse itesngsssm asvcgaslal mdagvpikaa vagiamglvk egdnyvvlsd
481 ilgdedhlgd mdfkvagsrd gisalqmdik iegitkeimq valnqakgar lhilgvmeqa
541 inaprgdise faprihtiki npdkikdvig kggsviralt eetgttieie ddgtvkiaat
601 dgekakhair rieeitaeie vgrvytgkvt rivdfgafva igggkeglvh isqiadkrve
661 kvtdylqmgq evpvkvlevd rqgrirlsik eateqsqpaa apeapaaeqg e

Mutation with R93 and R97

1 mlnpivrkfq ygqhtvtlet gmmarqataa vmvsmddtav fvtvvgqkka kpgqdffplt
61 vnyqertyaa gripgsffrr egrpsegetl iaglidgpir plfpegfvne vqviatvvsv
121 npqvnpdiva migasaalsl sgipfngpig aarvgyindq yvlnptqdel keskldlvva
181 gteaavlmve seaqllsedq mlgavvfghe qqqvviqnin elvkeagkpr wdwqpepvne
241 alnarvaala earlsdayri tdkqeryaqv dviksetiat llcedetlde nelgeilhai
301 eknvvrsrvl agepridgre kdmirgldvr tgvlprthgs alftrgetqa lvtatlgtar
361 daqvldelmg ertdtflfhy nfppysvget gmvgspkrre ighgrlakrg vlavmpdmdk
421 fpytvrvvse itesngsssm asvcgaslal mdagvpikaa vagiamglvk egdnyvvlsd
481 ilgdedhlgd mdfkvagsrd gisalqmdik iegitkeimq valnqakgar lhilgvmeqa
541 inaprgdise faprihtiki npdkikdvig kggsviralt eetgttieie ddgtvkiaat
601 dgekakhair rieeitaeie vgrvytgkvt rivdfgafva igggkeglvh isqiadkrve
661 kvtdylqmgq evpvkvlevd rqgrirlsik eateqsqpaa apeapaaeqg e

Supplementary Note 5. Systematic analysis of statistical data from the RNA analog binding process.

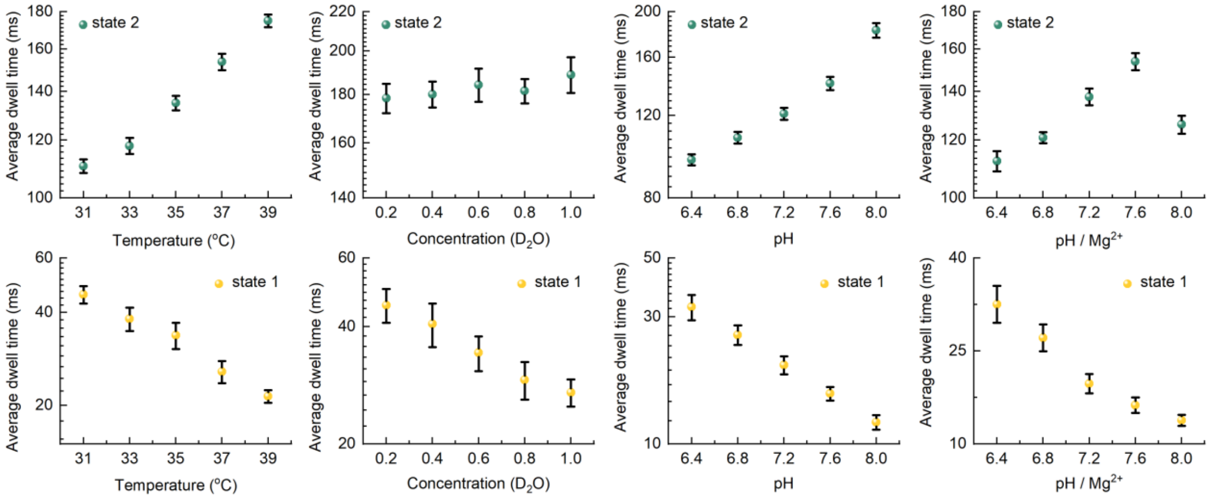

Supplementary Fig. 5 | Dwell time distributions of the low conductance state (orange, State 1) and the intermediate state (green, State 2) in temperature, RNA analog concentration, pH and pH–Mg^{2+} gradient experiments (2 mmol•L^{-1} $MgCl_2$) during the binding process (mean of $n = 3$ technical replicates from three different single-PNPase-modified SiNW devices, error bars indicate s.d.).

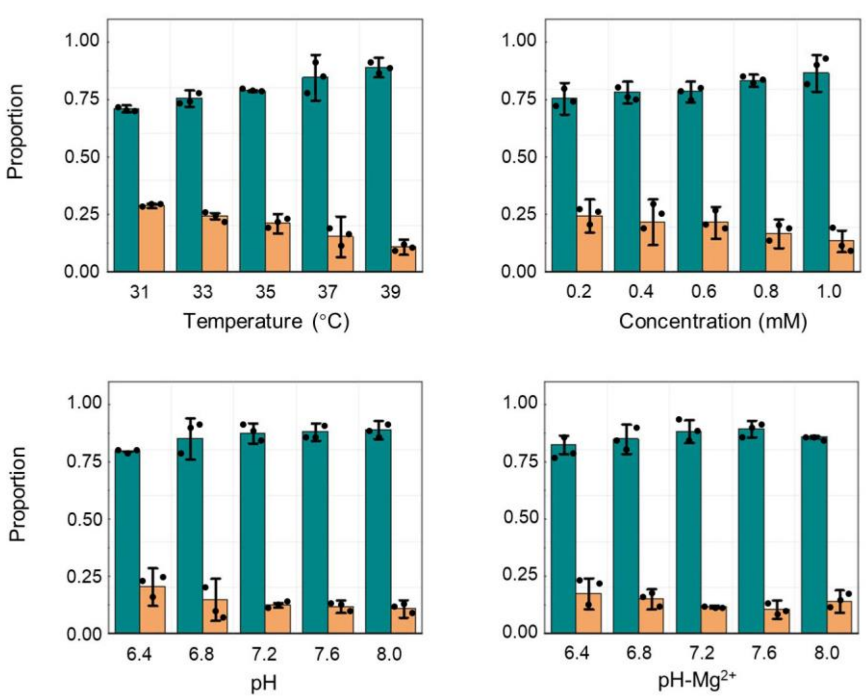

Supplementary Fig. 6 | Occurrence proportion distributions of the low conductance state (orange) and the intermediate state (green) in temperature, RNA analog concentration, pH and pH–Mg^{2+} gradient experiments (2 mmol•L^{-1} $MgCl_2$) during the binding process (mean of $n = 3$ technical replicates from three different single-PNPase-modified SiNW devices, error bars indicate s.d.).

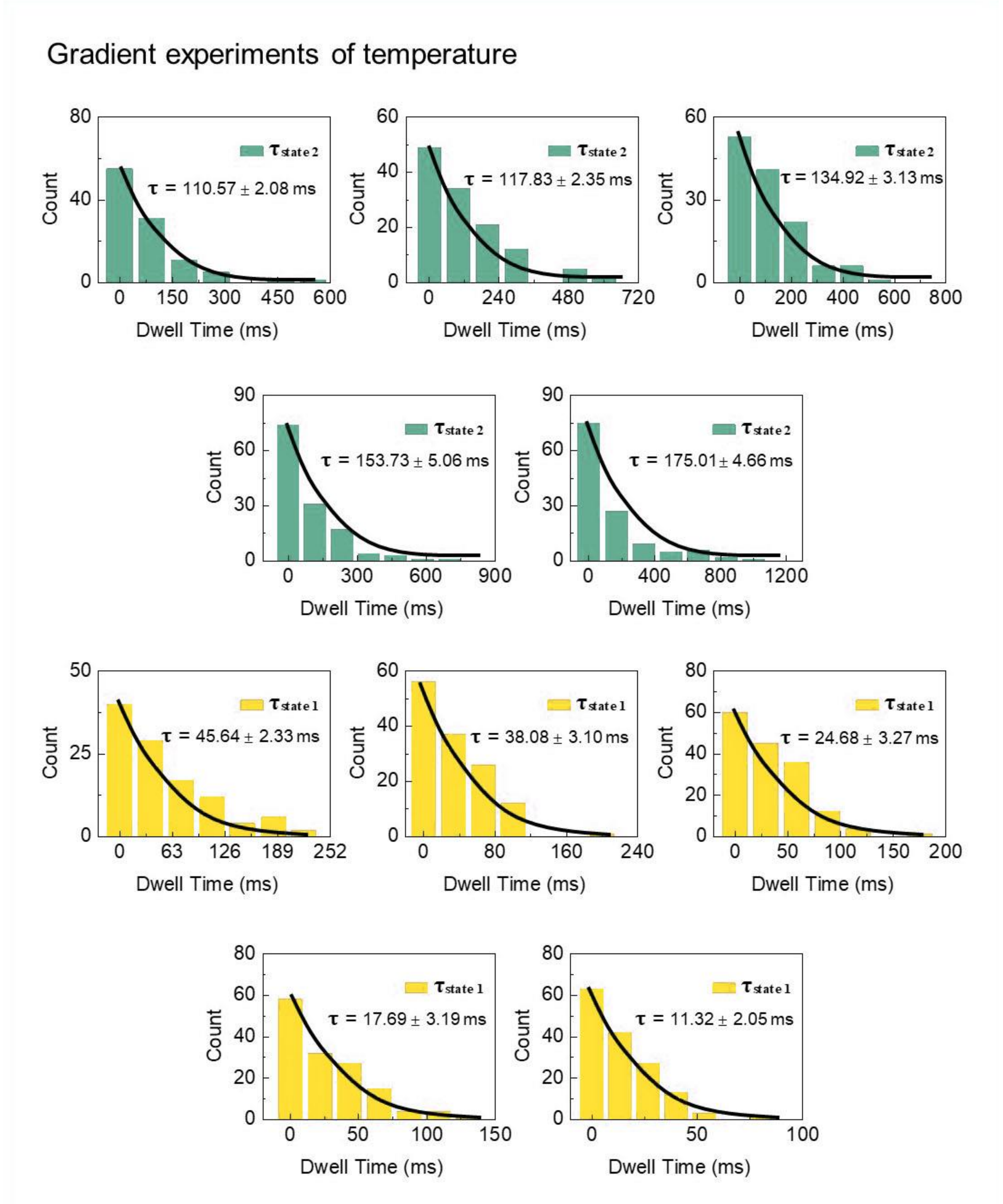

Supplementary Fig. 7 | Statistical analysis of the dwell times belonging to the low state (yellow) and intermediate state (green) in temperature gradient experiments (from upper left to bottom right: from 31 °C to 39 °C at 2 °C interval) during the binding process.

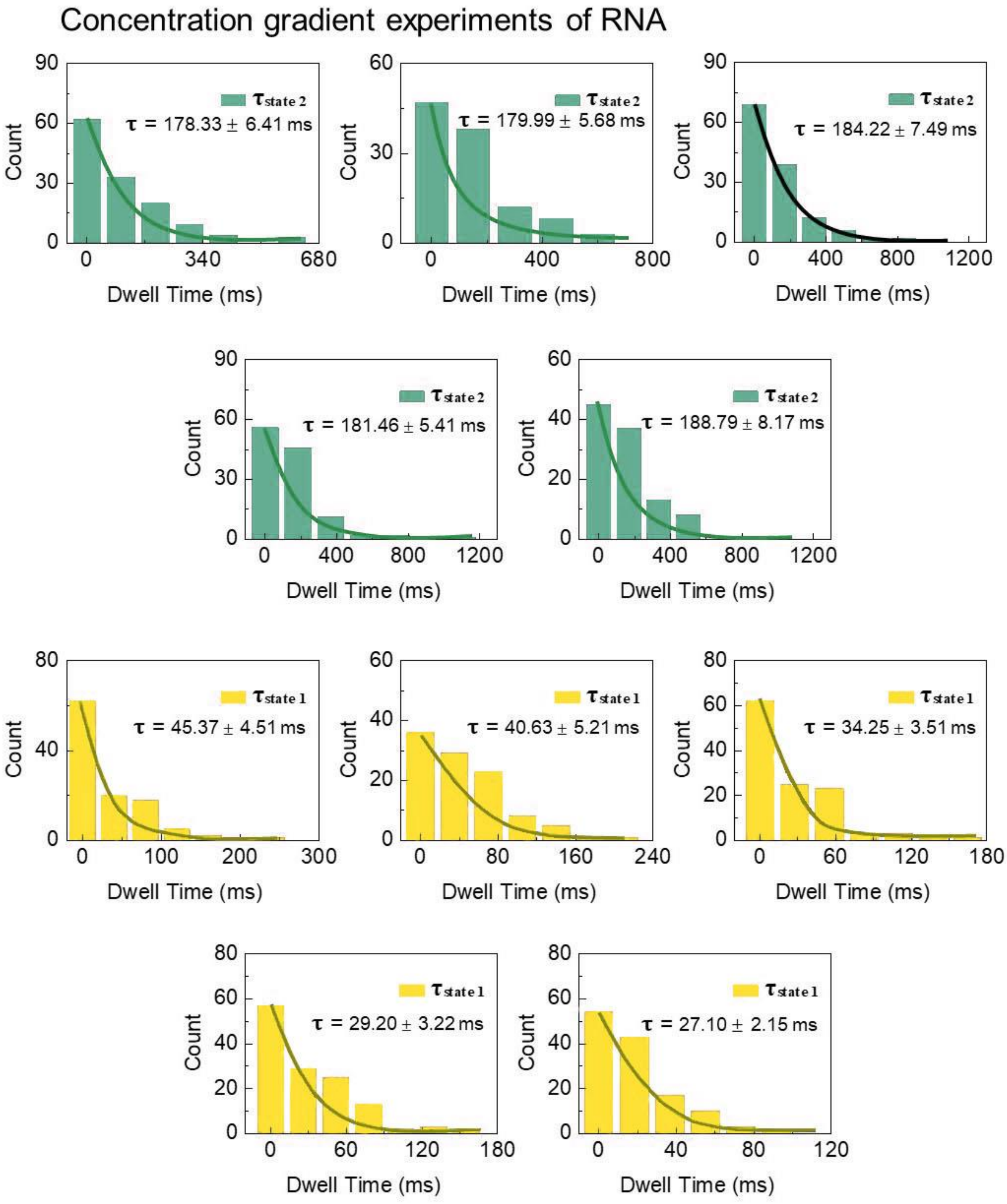

Supplementary Fig. 8 | Statistical analysis of the dwell times belonging to the low state (yellow) and intermediate state (green) in RNA analog concentration gradient experiments (from upper left to bottom right: from 0.2 mM•L^{-1} to 1 mM•L^{-1} at 0.2 mM•L^{-1} interval) during the binding process.

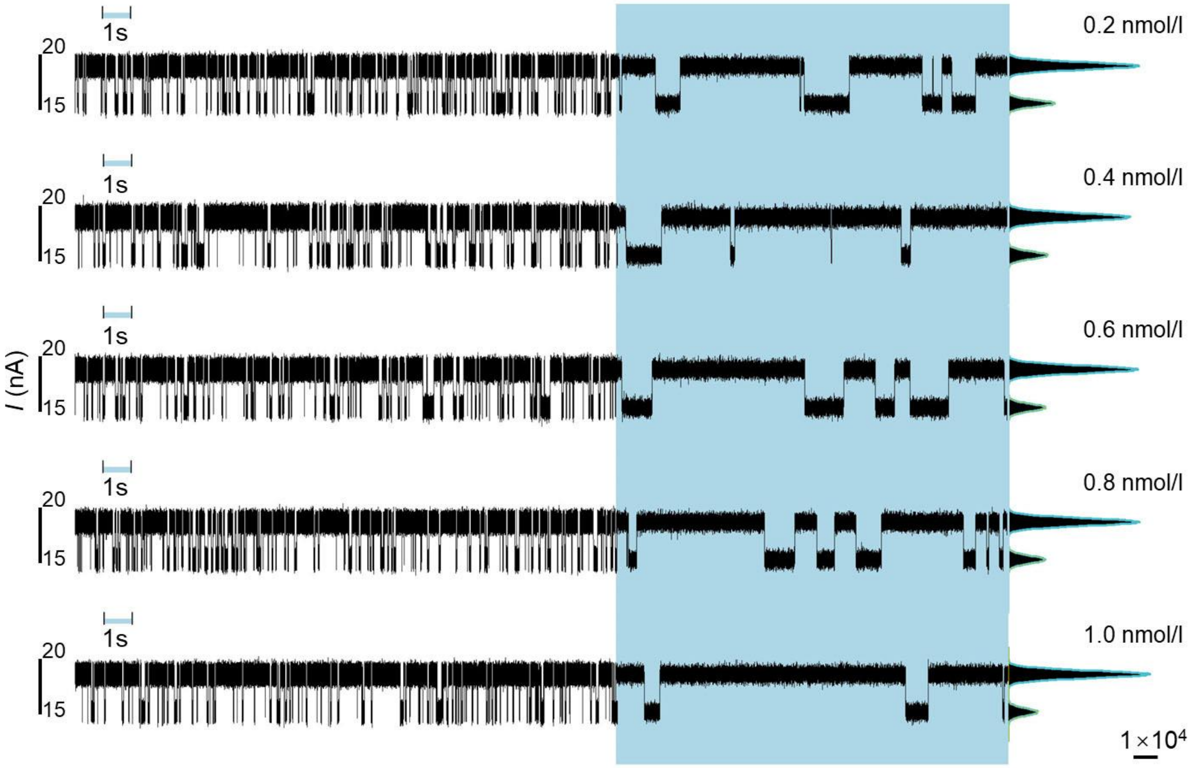

Supplementary Fig. 9 | Real–time electrical trajectories of the binding process between PNPase and RNA analog (poly(A_{30})) in concentration gradient experiments of the substrate (37 ºC, pH 7.6 and 2 mmol•L^{-1} $MgCl_2$). The middle panels (marked with cyan) showed the 1 s magnified view of each trace and the right panels were the corresponding current histograms of each trace.

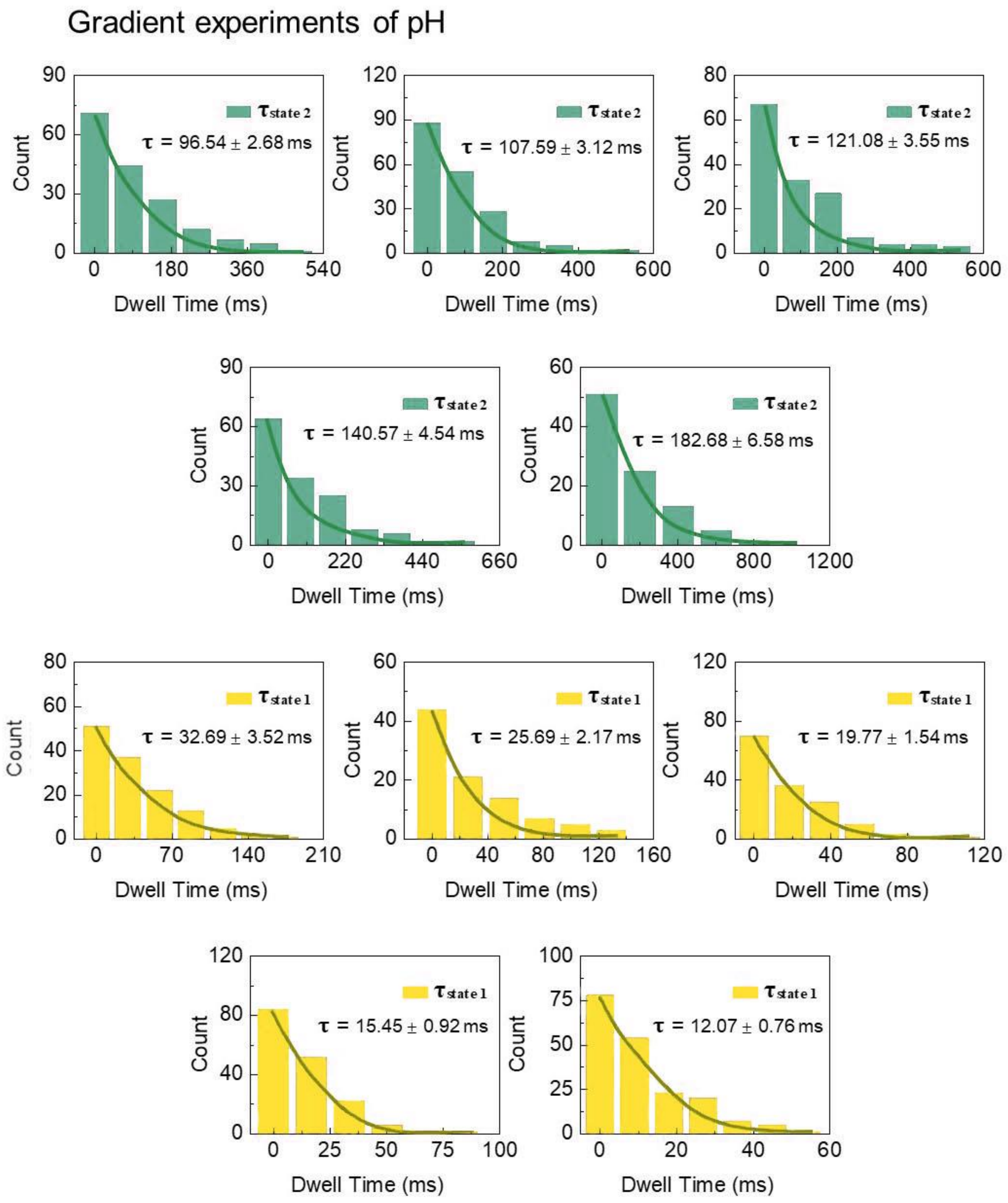

Supplementary Fig. 10 | Statistical analysis of the dwell times belonging to the low state (yellow) and intermediate state (green) in pH gradient experiments (from upper left to bottom right: from 6.4 to 8.0 at 0.4 intervals) during the binding process.

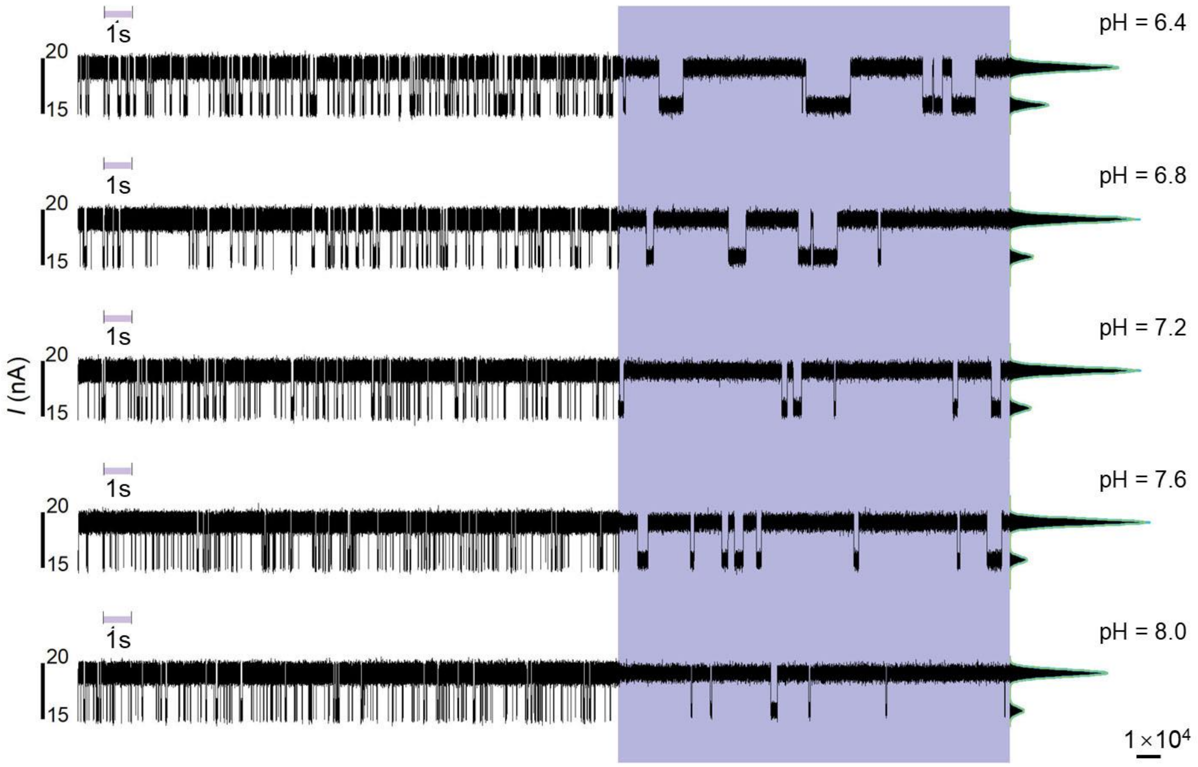

Supplementary Fig. 11 | Real–time electrical trajectories of the binding process between PNPase and RNA analog (poly(A_{30})) in pH gradient experiments (37°C, 1 mmol•L^{-1} RNA analog). The middle panels (marked with purple) show the 1 s magnified view of each trace and the right panels are the corresponding current histograms of each trace.

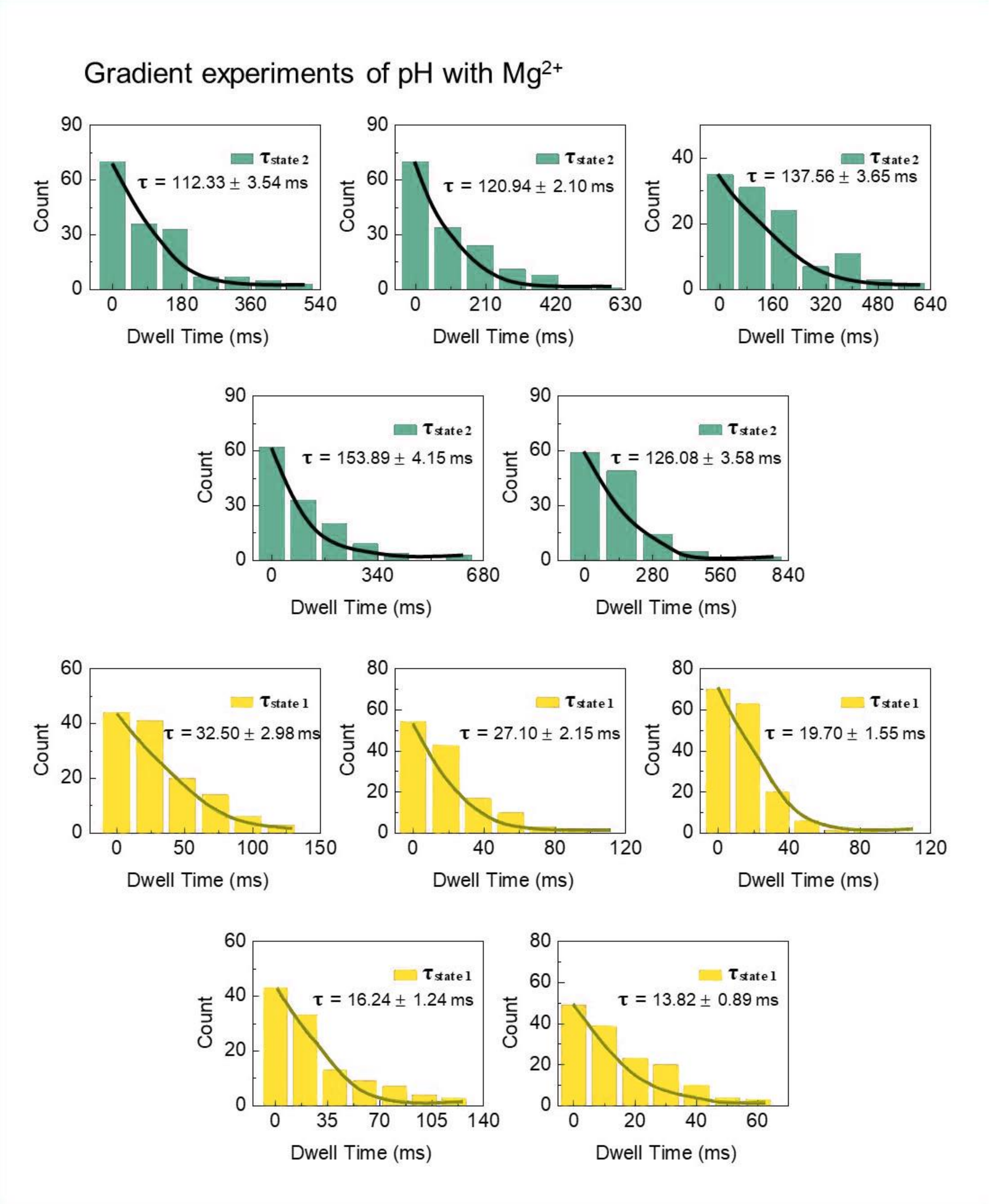

Supplementary Fig. 12 | Statistical analysis of the dwell times belonging to the low state (yellow) and intermediate state (green) in pH gradient experiments with Mg^{2+} (from upper left to bottom right: from 6.4 to 8.0 at 0.4 intervals; 2 mmol•L^{-1} $MgCl_2$) during the binding process.

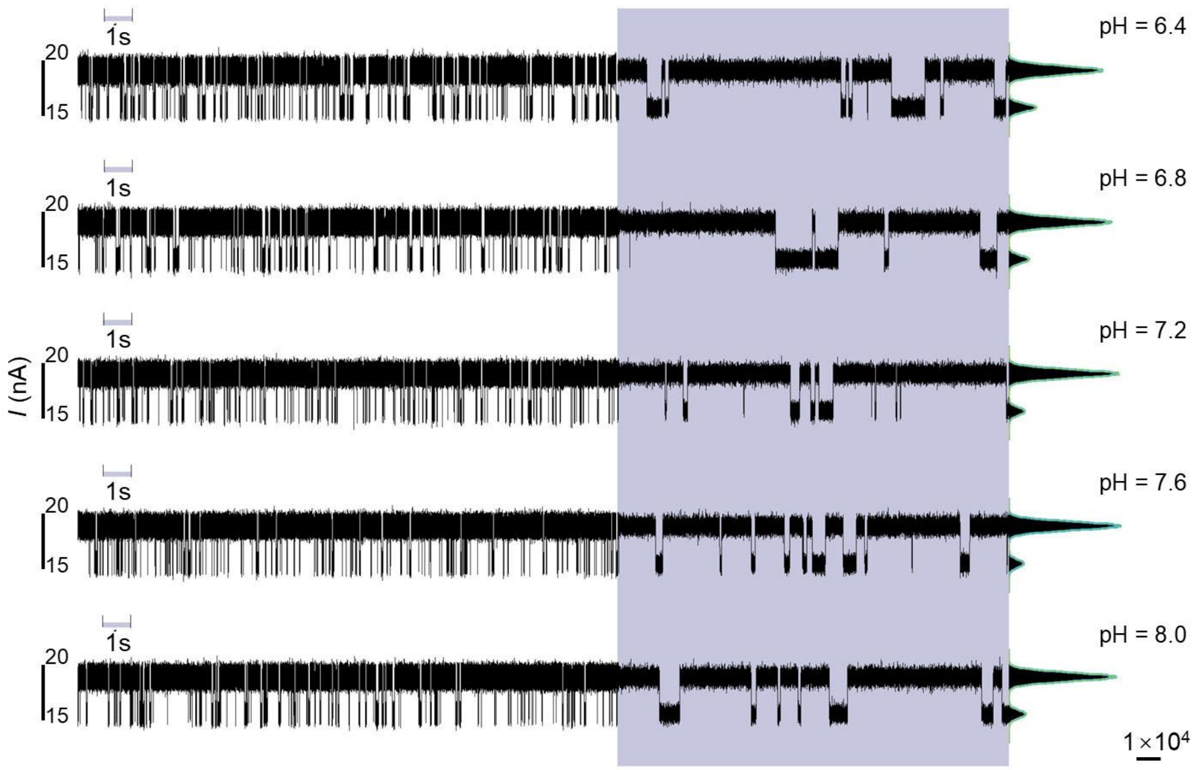

Supplementary Fig. 13 | Real–time electrical trajectories of the binding process between PNPase and RNA analog (poly(A_{30})) in pH gradient experiments (37°C, 1 mmol•L^{-1} RNA analog, 2 mmol•L^{-1} $MgCl_2$). The middle panels (marked with dark purple) show the 1 s magnified view of each trace and the right panels are the corresponding current histograms of each trace.

Supplementary Note 6. Systematic analysis of statistical data from the RNA analog degradation process.

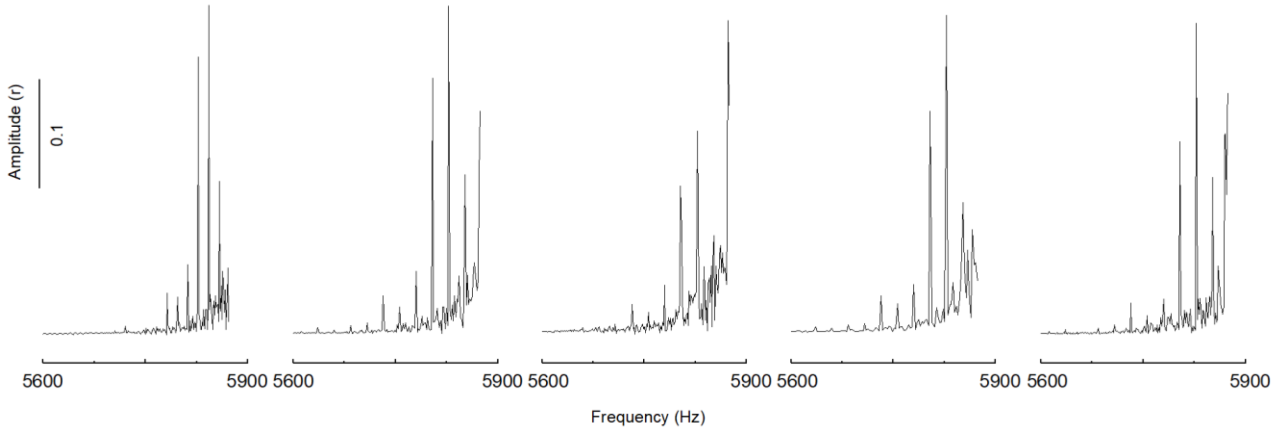

Supplementary Fig. 14 | Constant high–frequency vibrations were assigned to the apparatus signal noise.

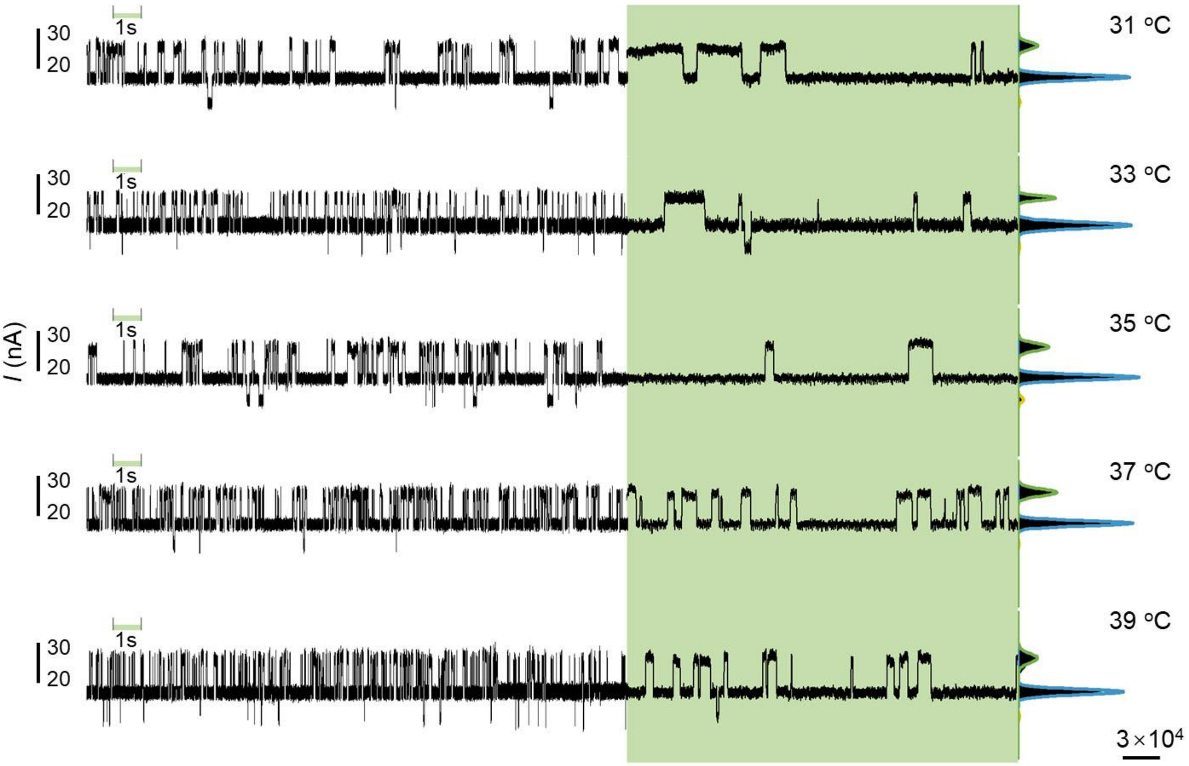

Supplementary Fig. 15 | Real–time electrical trajectories of the degradation process of RNA analog (poly(A_{30})) in temperature gradient experiments with Mn^{2+} (1 mmol•L^{-1} RNA analog, pH 7.6, 2 mmol•L^{-1} $MnCl_2$ and H_3PO_4 8 mM•L^{-1}). The middle panels (marked with green) show the 1 s magnified view of each trace and the right panels are the corresponding current histograms of each trace.

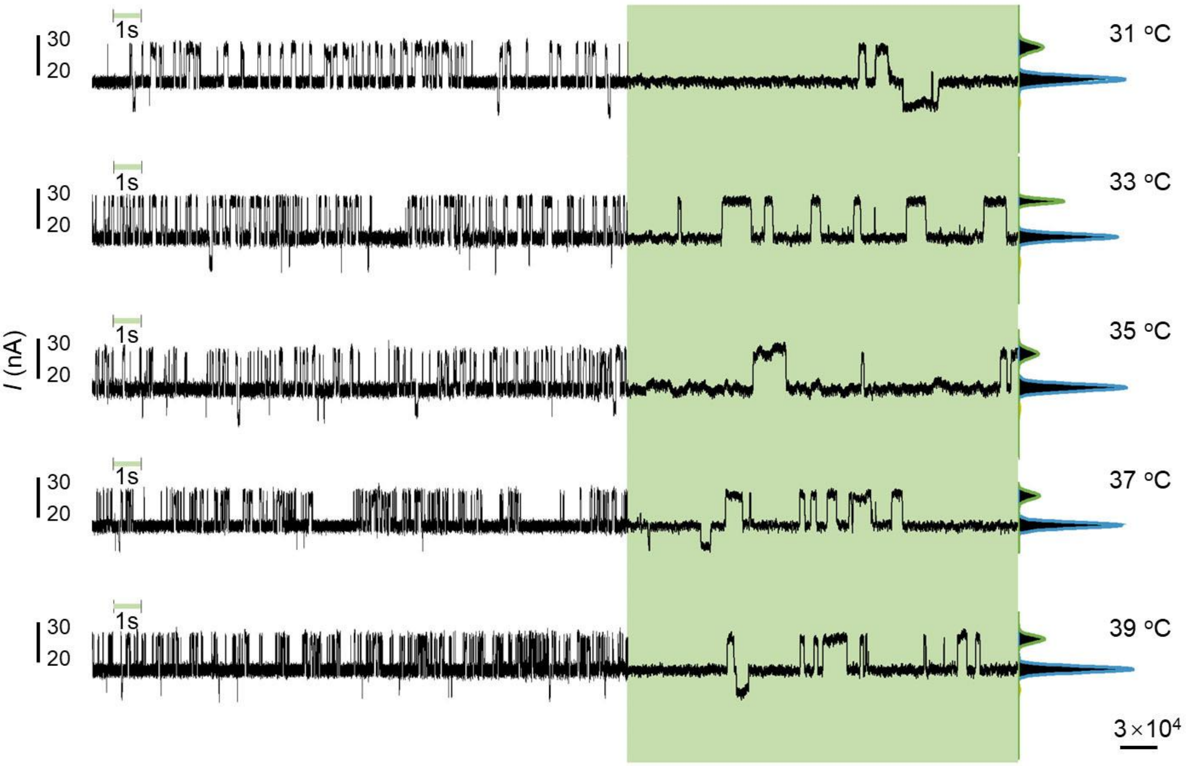

Supplementary Fig. 16 | Real–time electrical trajectories of the degradation process of RNA analog (poly(A_{30})) in temperature gradient experiments with D_2O (1 mmol•L^{-1} RNA analog, pH 7.6, 2 mmol•L^{-1} $MgCl_2$ and H_3PO_4 8 mM•L^{-1}). The middle panels (marked with green) show the 1 s magnified view of each trace and the right panels are the corresponding current histograms of each trace.

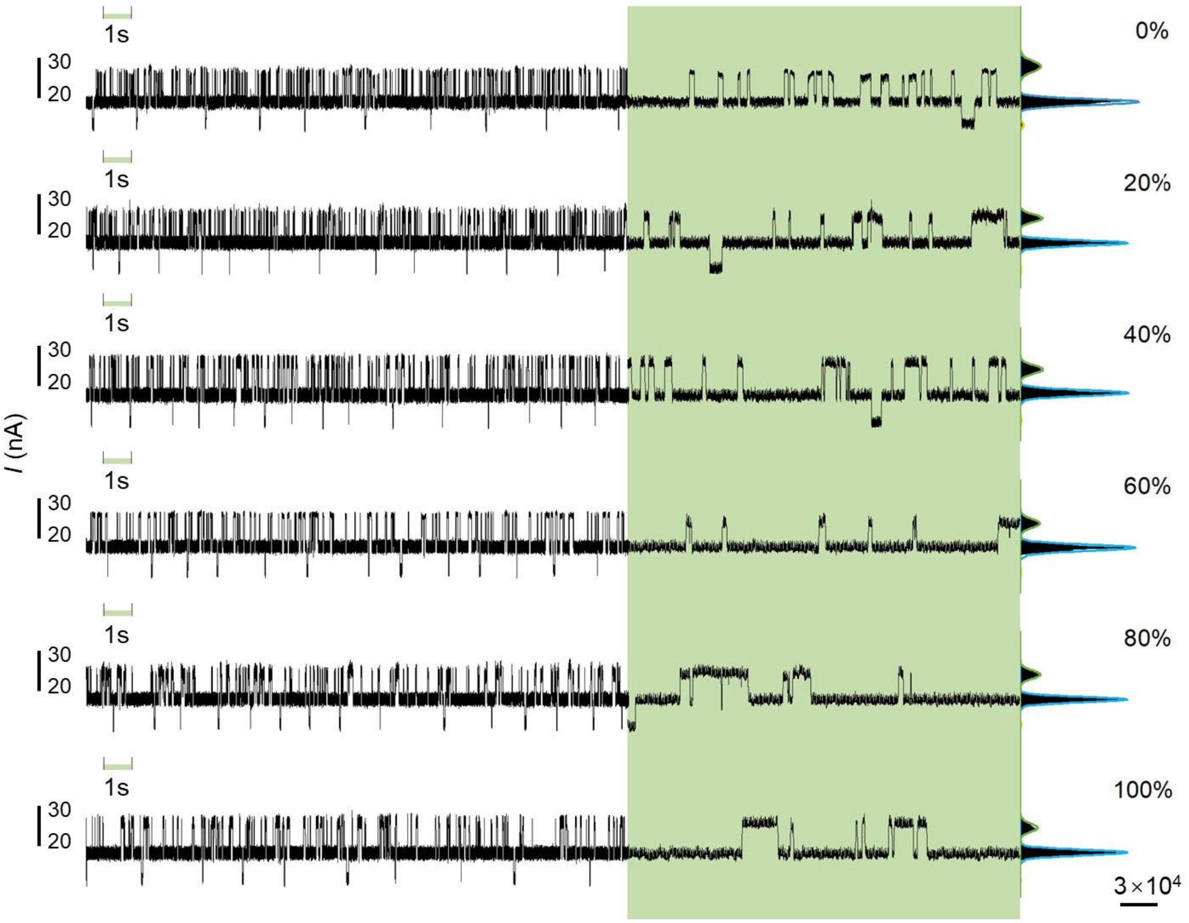

Supplementary Fig. 17 | Real–time electrical trajectories of the degradation process of RNA analog (poly(A_{30})) in concentration gradient experiments of D_2O (37 °C, 1 mmol•L^{-1} RNA analog, pH 7.6, 2 mmol•L^{-1} $MgCl_2$ and H_3PO_4 8 mM•L^{-1}). The middle panels (marked with green) show the 1 s magnified view of each trace and the right panels are the corresponding current histograms of each trace.

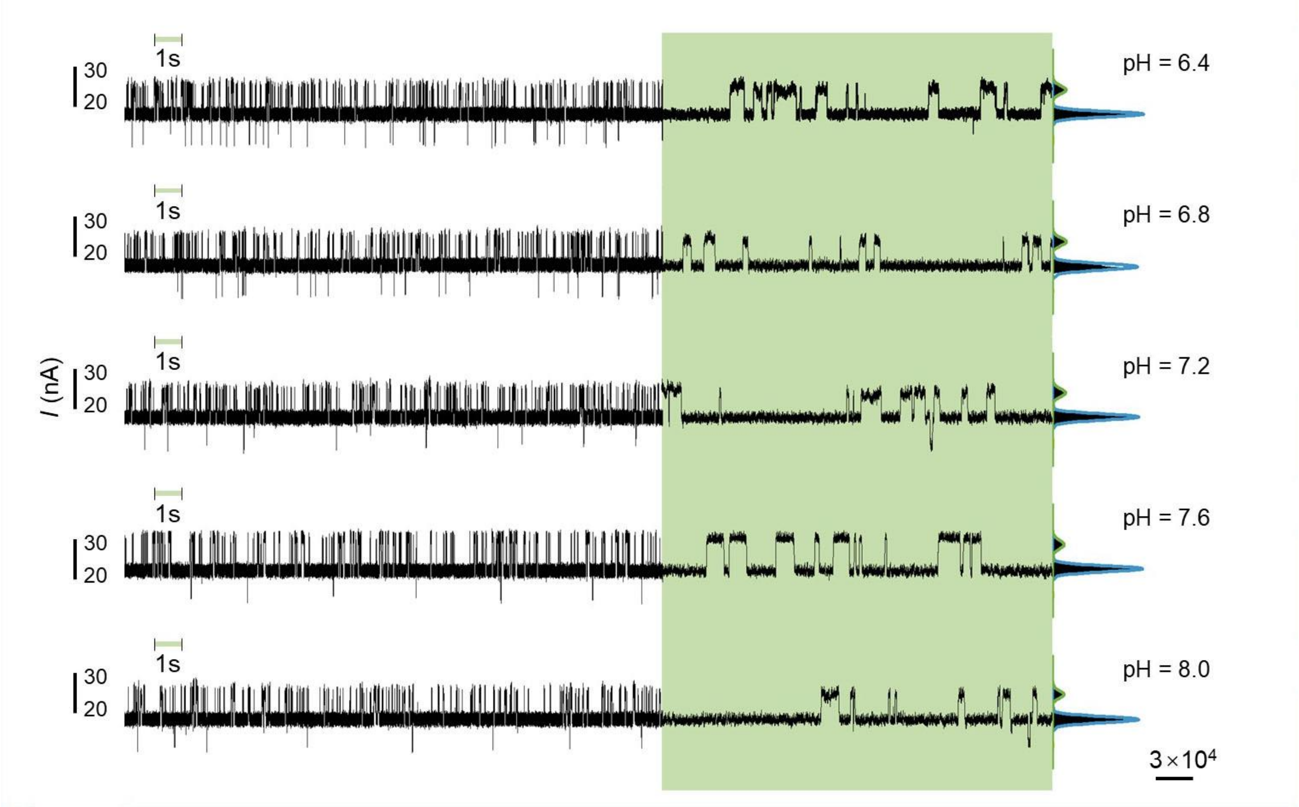

Supplementary Fig. 18 | Real–time electrical trajectories of the degradation process of RNA analog (poly(A_{30})) in pH gradient experiments (37 °C, 1 mmol•L^{-1} RNA analog, 2 mmol•L^{-1} $MgCl_2$ and H_3PO_4 8 mM•L^{-1}). The middle panels (marked with green) show the 1 s magnified view of each trace and the right panels are the corresponding current histograms of each trace.

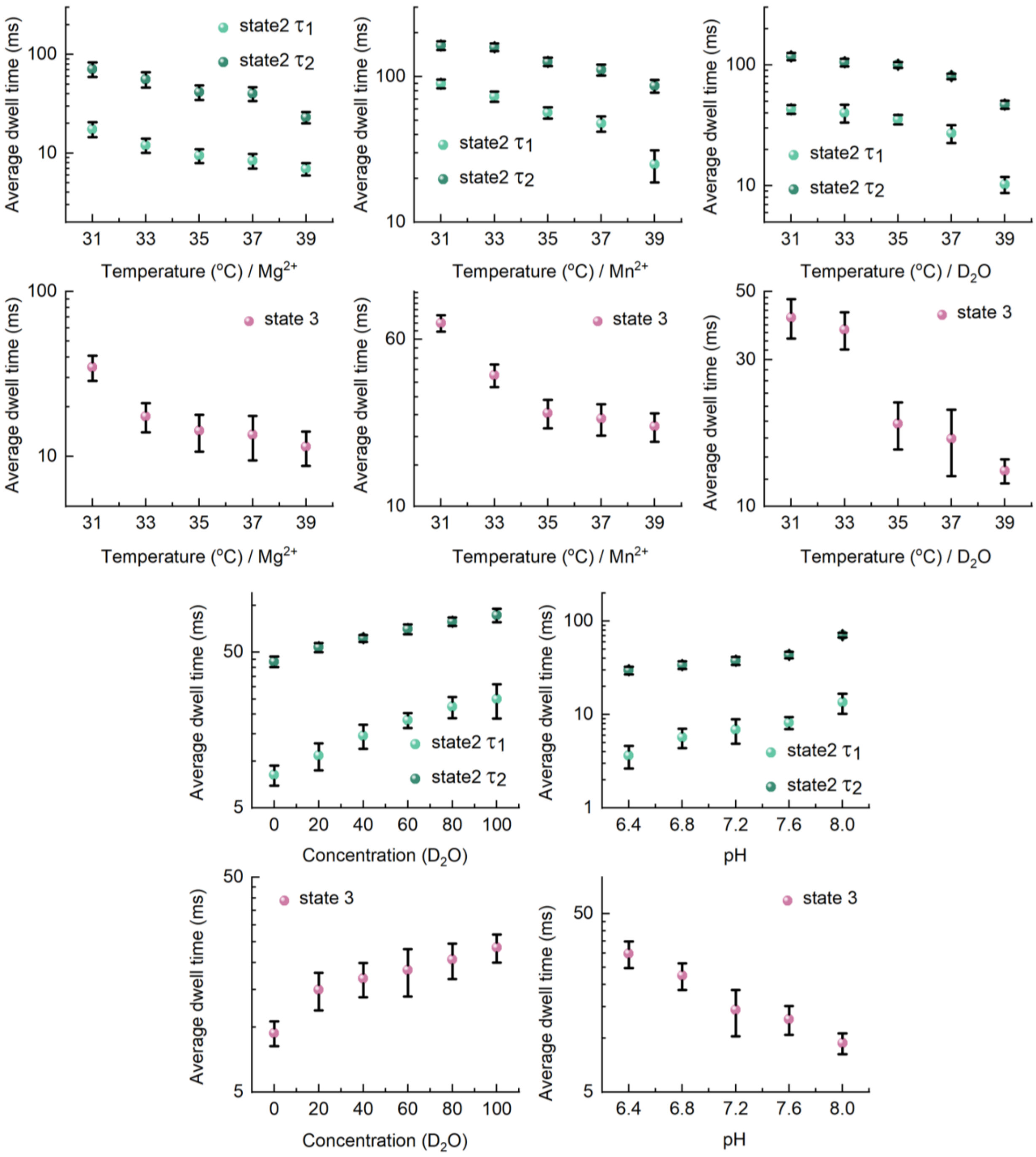

Supplementary Fig. 19 | Dwell time distributions of the intermediate state (green) and the high state (purple) in D_2O gradient concentration experiments at 37 ℃, temperature gradient experiments in the presence of Mg^{2+} (2 mmol•L^{-1} $MgCl_2$), Mn^{2+} (2 mmol•L^{-1} $MnCl_2$), D_2O (solution volume percent 100%) and pH gradient experiments at 37 ℃ during the degradation process (mean of n = 3 technical replicates from three different single-PNPase-modified SiNW devices, error bars indicate s.d.).

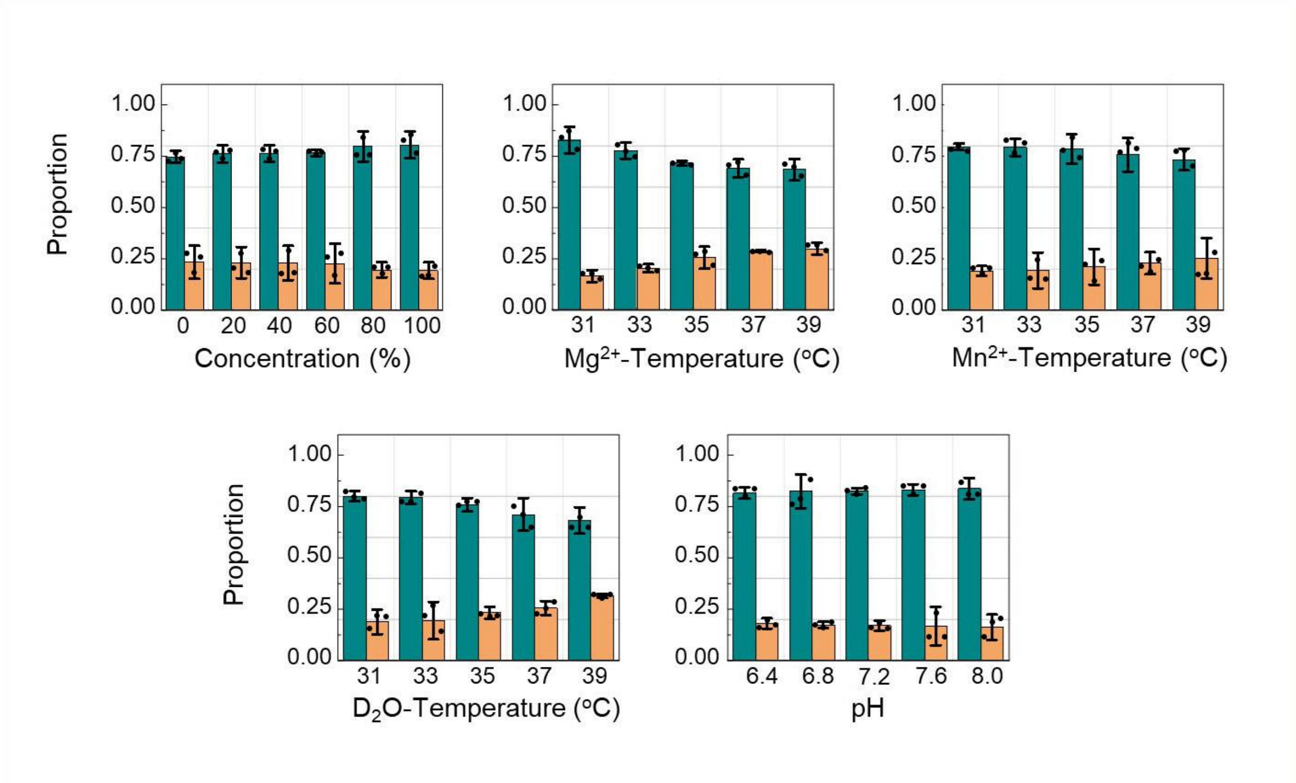

Supplementary Fig. 20 | Occurrence proportion distributions of the intermediate state (green) and the high state (orange) in D_2O gradient concentration experiments at 37 °C, temperature gradient experiments in the presence of Mg^{2+} (2 mmol•L^{-1} $MgCl_2$), Mn^{2+} (2 mmol•L^{-1} $MnCl_2$), D_2O (solution volume percent 100%) and pH gradient experiments at 37 °C during the degradation process (mean of $n = 3$ technical replicates from three different single-PNPase-modified SiNW devices, error bars indicate s.d.).

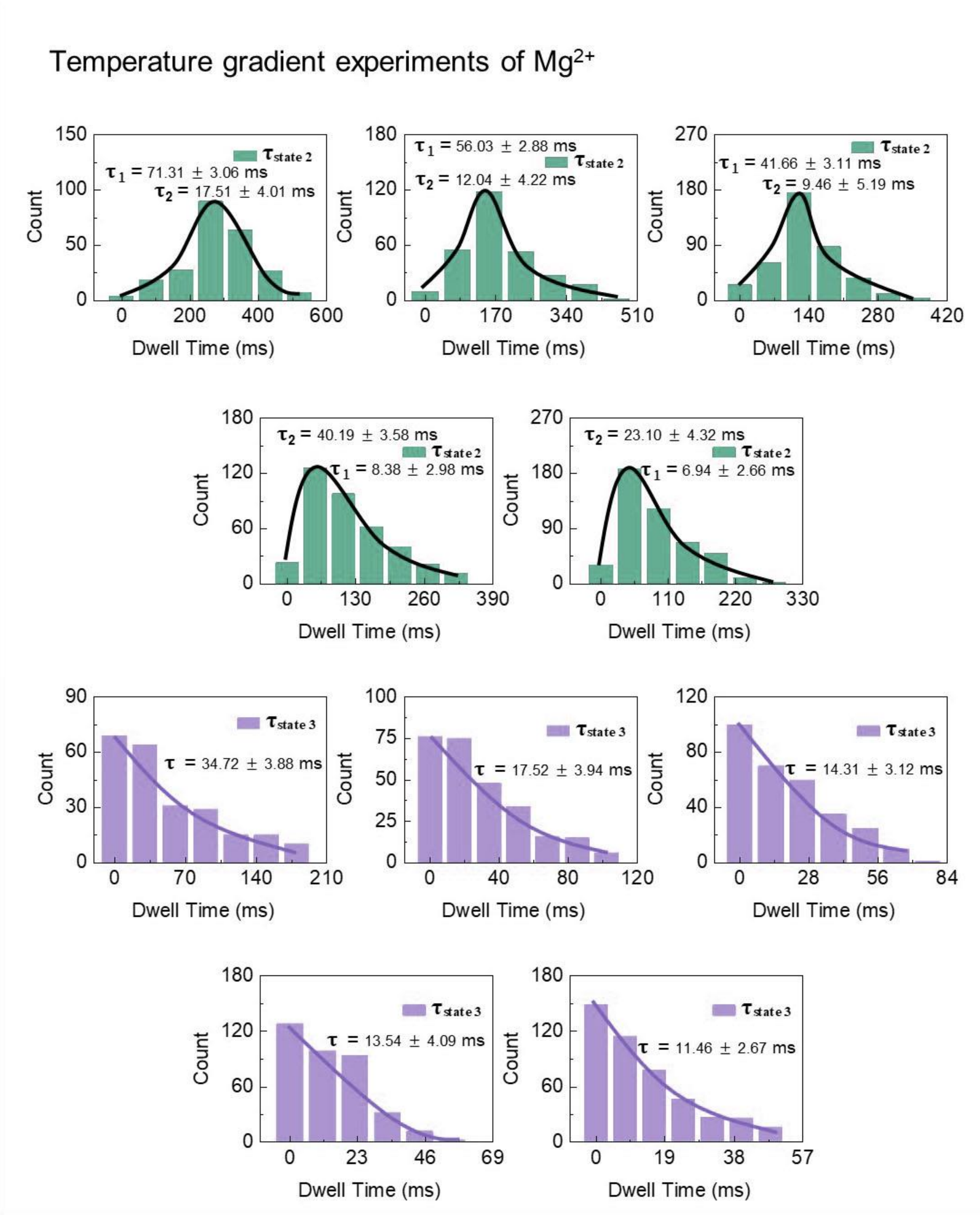

Supplementary Fig. 21 | Statistical analysis of the dwell times belonging to the intermediate state (green) and the high state (purple) in temperature gradient experiments with Mg^{2+} (from upper left to bottom right: from 31 °C to 39 °C at 2 °C interval; 2 mmol•L^{-1} $MgCl_2$) during the degradation process.

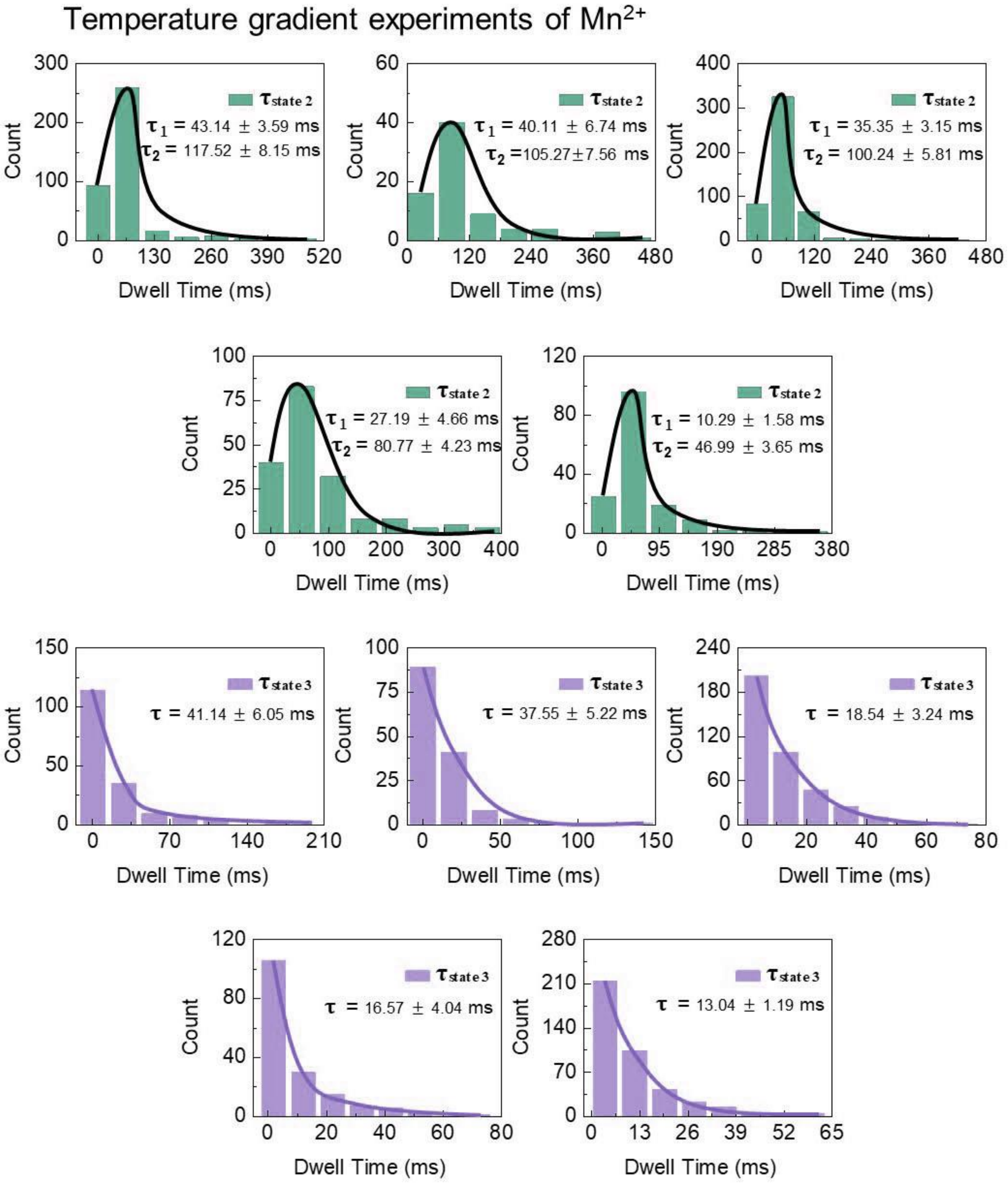

Supplementary Fig. 22 | Statistical analysis of the dwell times belonging to the intermediate state (green) and the high state (purple) in temperature gradient experiments with Mn^{2+} (from upper left to bottom right: from 31 ℃ to 39 ℃ at 2 ℃ interval; 2 mmol•L^{-1} $MnCl_2$) during the degradation process.

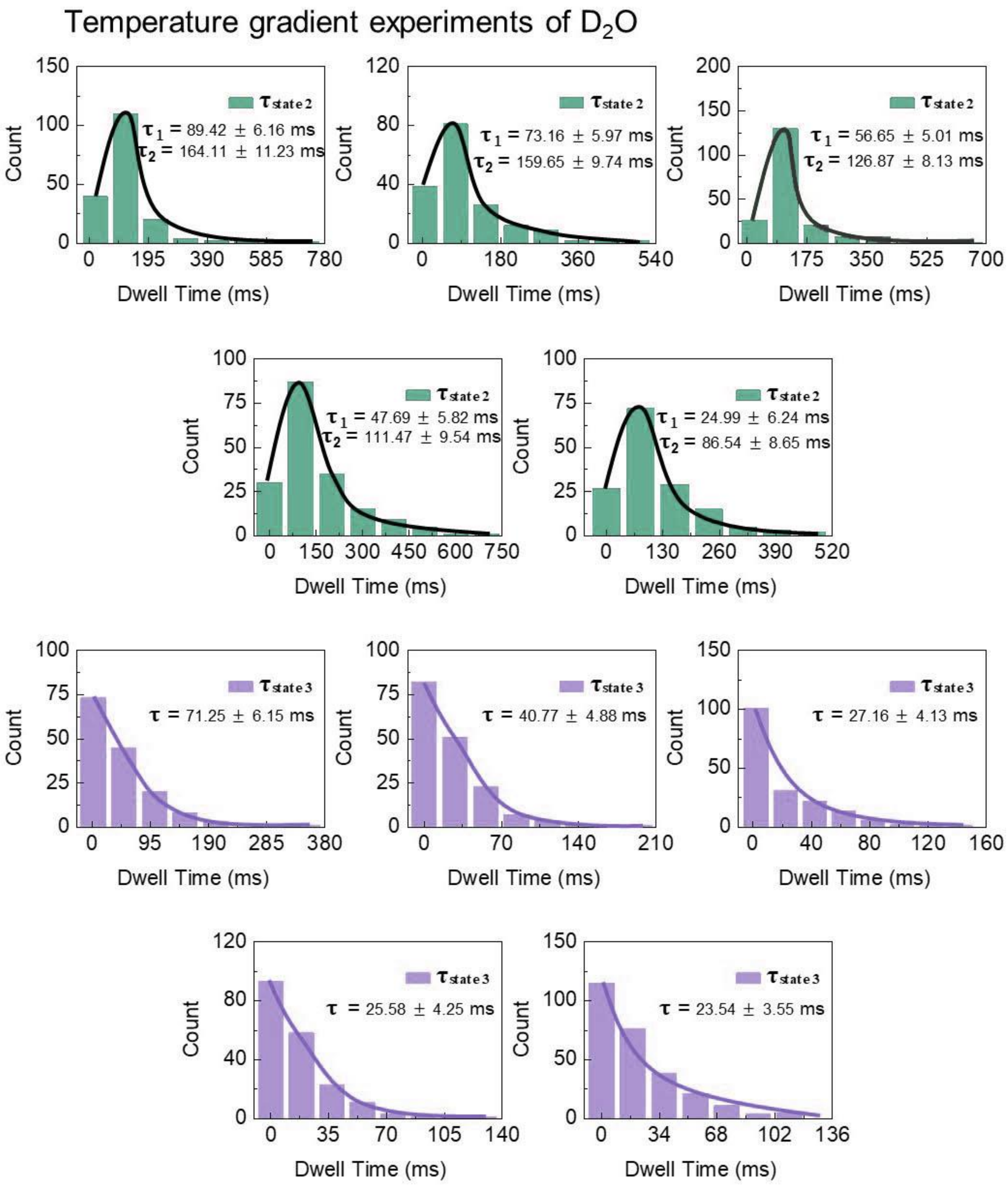

Supplementary Fig. 23 | Statistical analysis of the dwell times belonging to the intermediate state (green) and the high state (purple) in temperature gradient experiments with D_2O solution (from upper left to bottom right: from 31 ℃ to 39 ℃ at 2 ℃ interval; D_2O solution volume percent: 100%) during the degradation process.

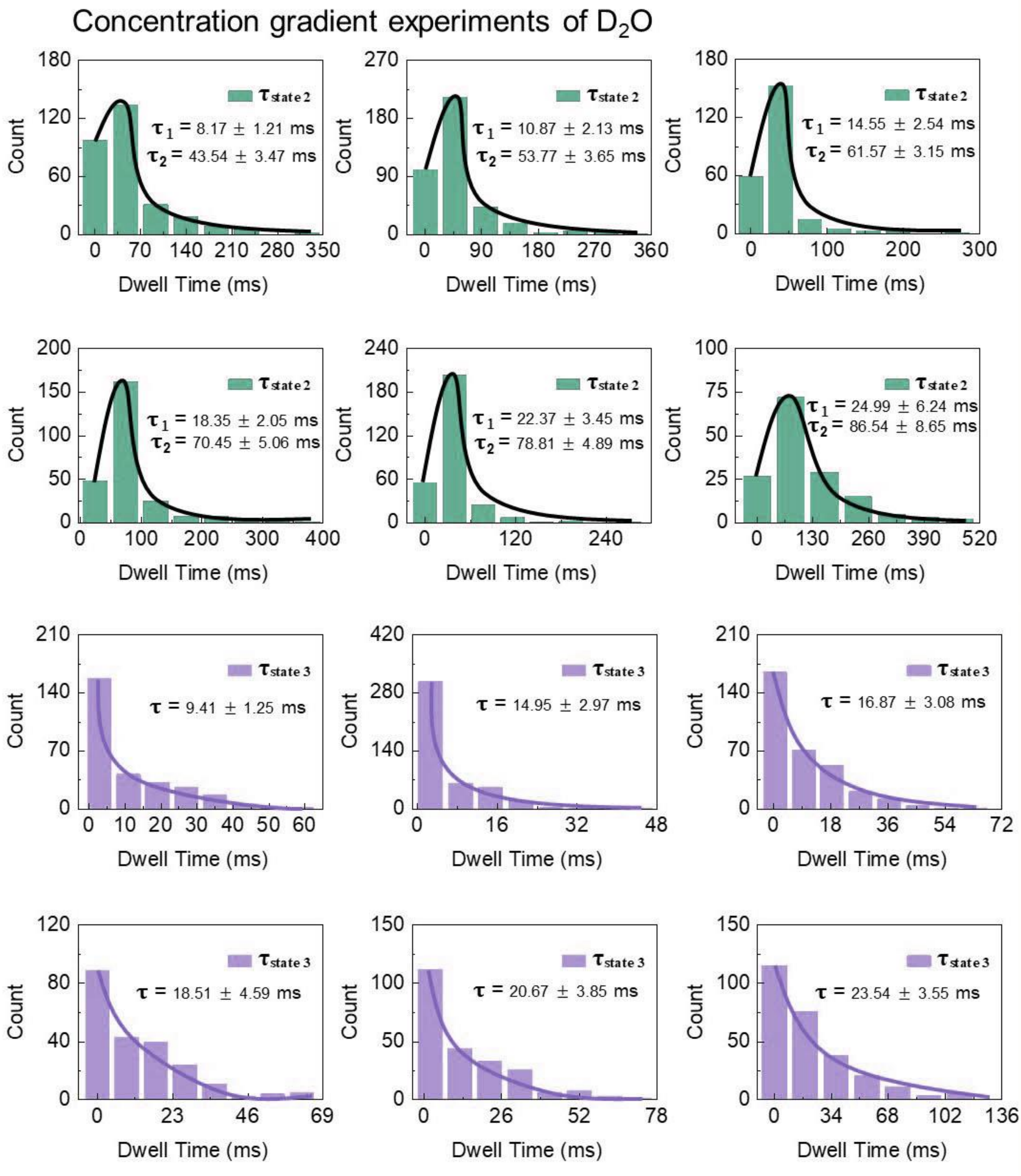

Supplementary Fig. 24 | Statistical analysis of the dwell times belonging to the intermediate state (green) and the high state (purple) in concentration gradient experiments with D_2O (from upper left to bottom right: from 0 % to 100 % (solution volume percent) at 20 % interval) during the degradation process.

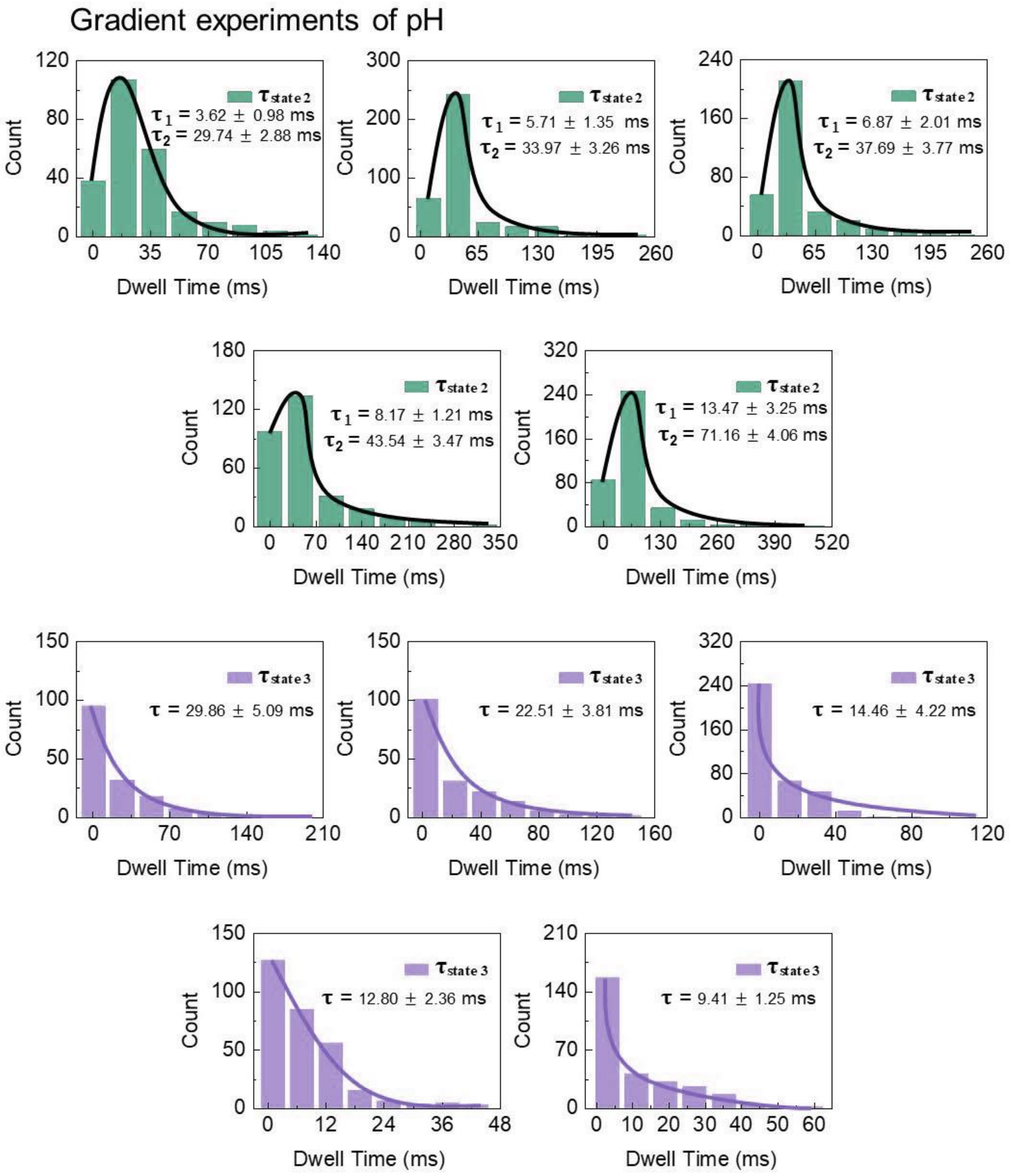

Supplementary Fig. 25 | Statistical analysis of the dwell times belonging to the intermediate state (green) and the high state (purple) in pH gradient experiments (from upper left to bottom right: from 6.4 to 8.0 at 0.4 intervals) during the degradation process.

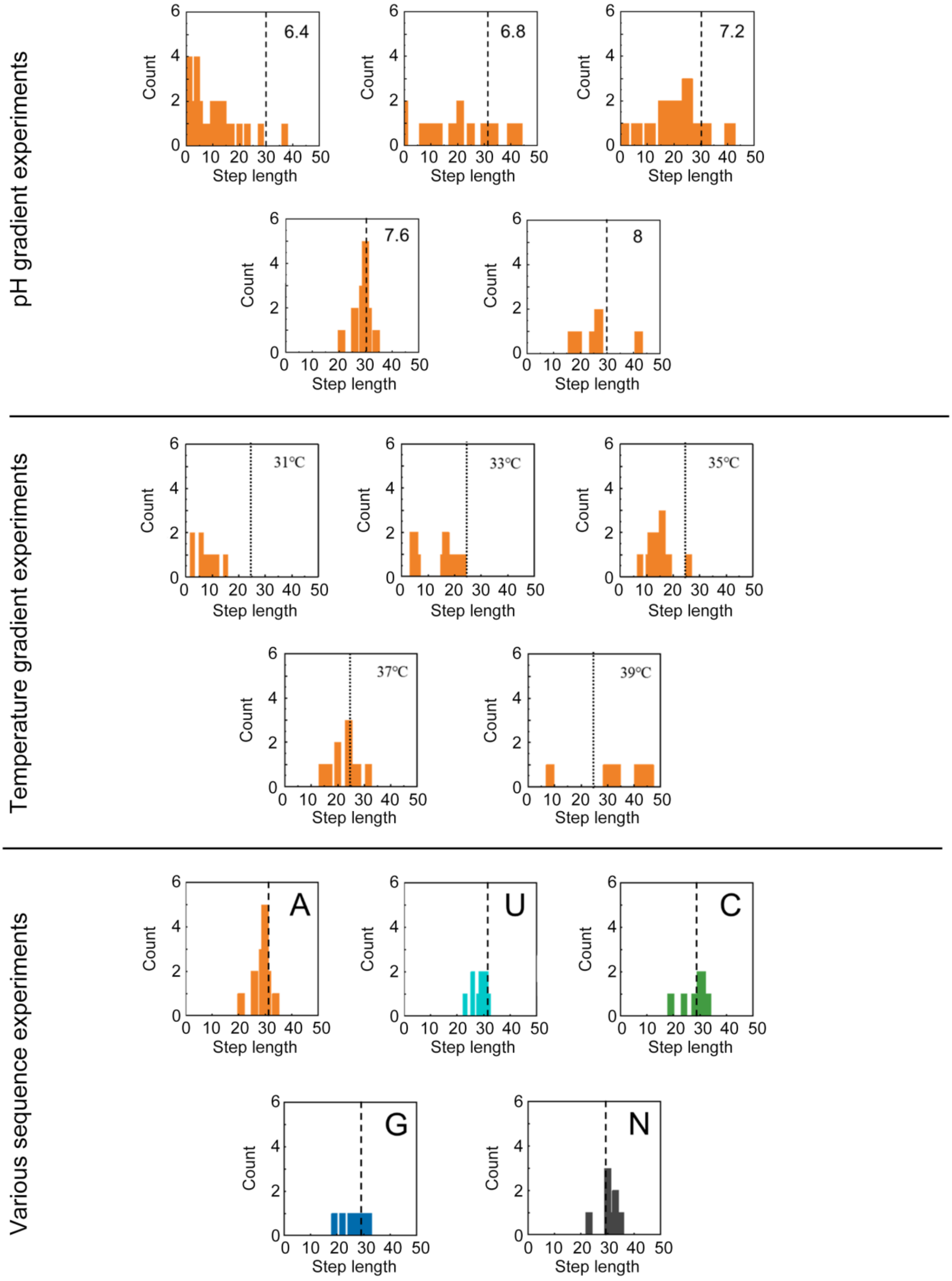

Supplementary Fig. 26 | The step-length counting of the degradation process at pH gradient experiments for ploy(A)$_{30}$ (up panel), at temperature gradient experiments for ploy(A)$_{30}$ (middle panel), and for various substrates (bottom panel).

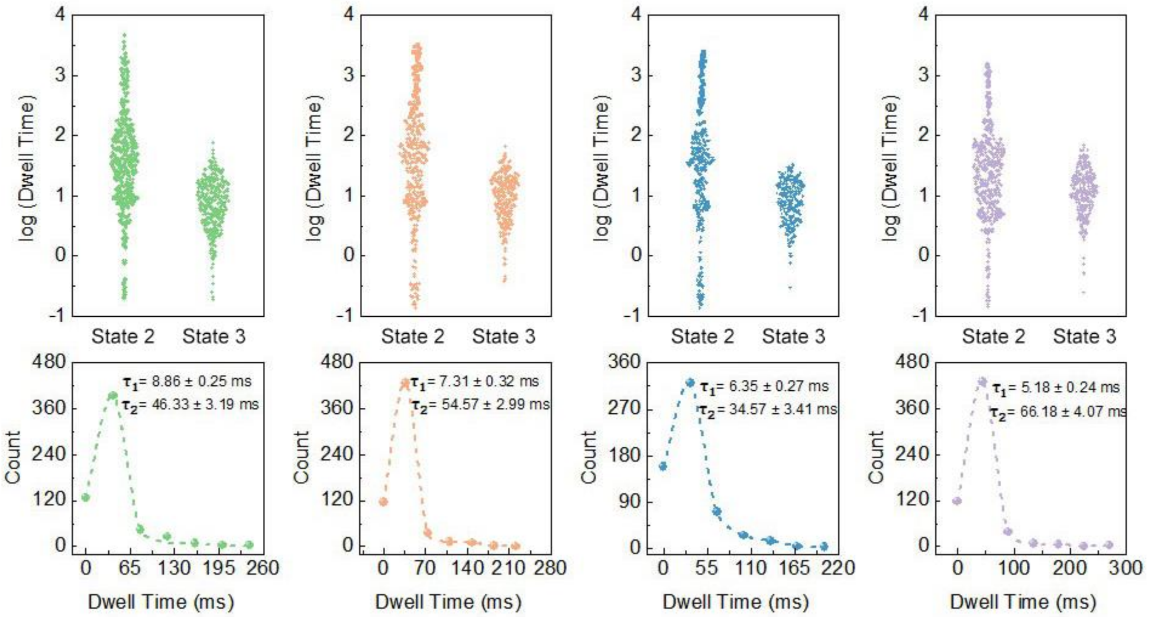

Supplementary Fig. 27 | Dwell time (ms) distribution (top panel) and statistical average dwell times (bottom panel) of the binding structure for four types of RNA analog with homogeneous nucleotide sequences (State 2: intermediate state; State 3: high state) (poly(A)$_{30}$–green; poly(U)$_{30}$–orange; poly(C)$_{30}$–blue; poly(G)$_{30}$–purple). n = 4 biologically independent samples examined over Y = 4 independent experiments.

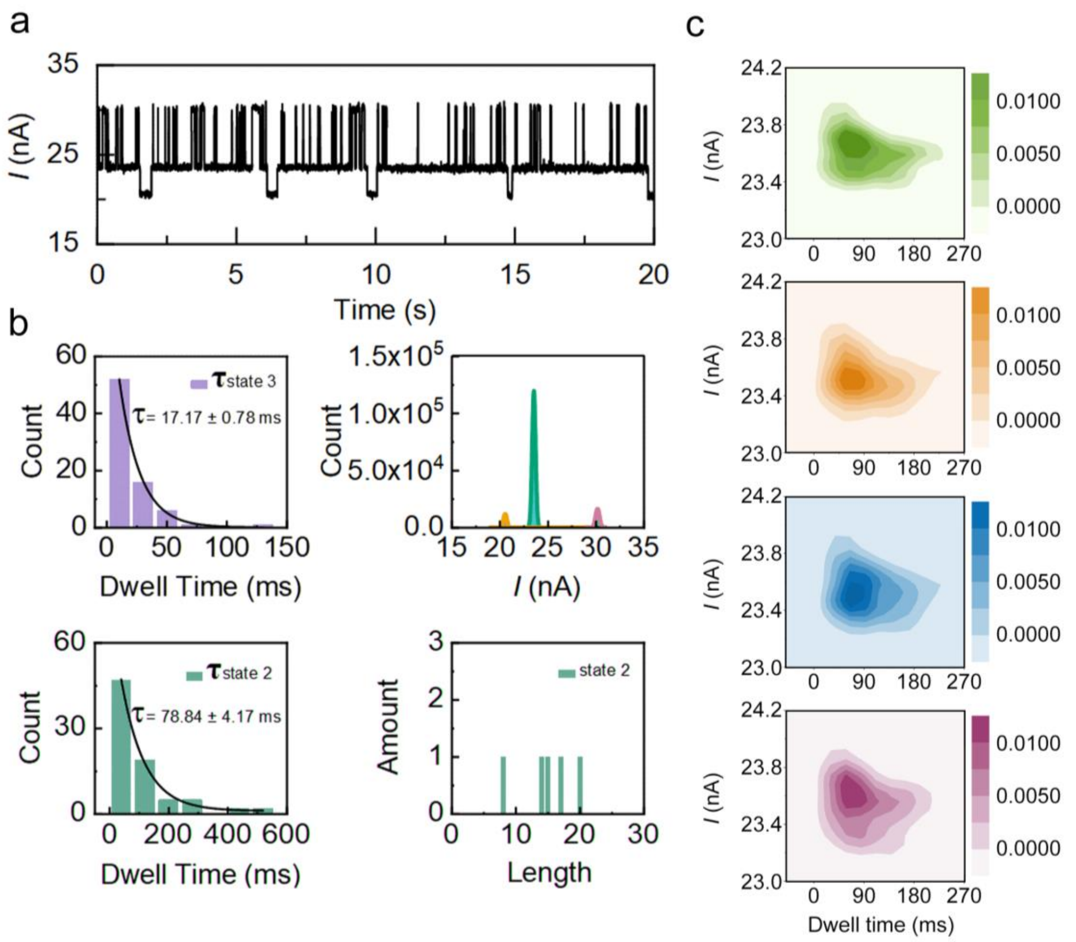

Supplementary Fig. 28 | Real–time electrical trajectories and statistical analysis of the mutant PNPase degradation process. (a) Real–time electrical trajectories of the mutant PNPase degradation process (37 °C, pH=7.6, 1 mmol•L^{-1} RNA analog, 2 mmol•L^{-1} $MgCl_2$ and H_3PO_4 8 mM•L^{-1}). (b) The single exponential fitting for the dwell time of the intermediate state (green) and high state (purple), the proportion distribution for three states (orange-low state, green-intermediate state, and purple-high state), and the step count distribution of the complete degradation process in 20 seconds. (c) The fingerprints of the intermediate state for four types of the homogenous sequence (poly(A)$_{30}$–green; poly(U)$_{30}$–orange; poly(C)$_{30}$–blue; poly(G)$_{30}$–purple).

Supplementary Note 7. Statistical analysis of fingerprint data from the degradation process of the heterogeneous sequence.

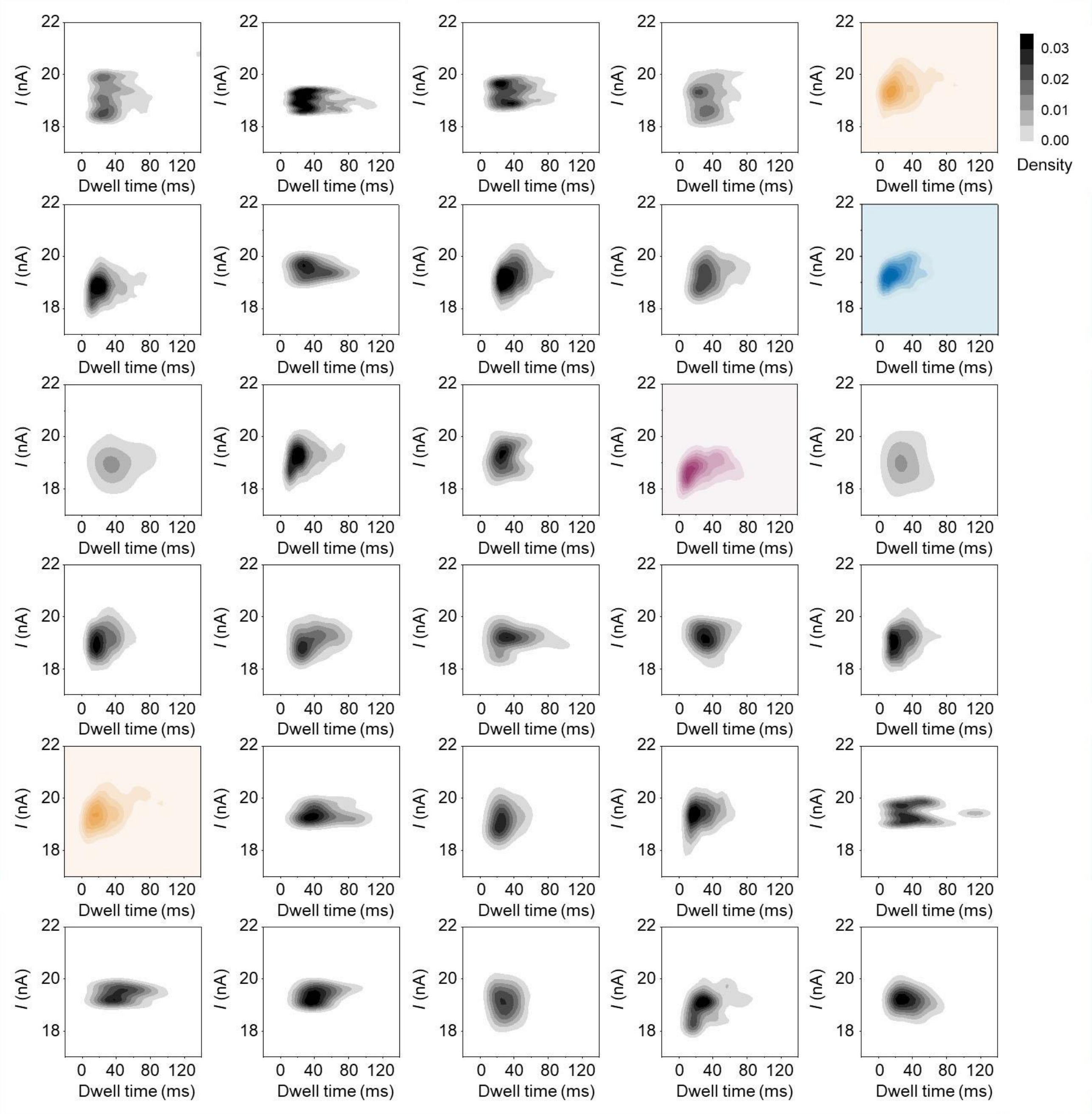

Supplementary Fig. 29 | Fingerprint map collection of 30 sites using a heterogeneous sequence (5'CGAUCUUCAUUGCCAAGCGGCUAGCUCAAA3'). Statical nucleotide information of heterogeneous sequence from upper left to bottom right: **5'**NNNNUNNNNNCNNNGNNNNNN UNNNNNNNNN**3'** (A–Adenine; U–Uracil; C–Cytosine; G–Guanine; N–Non-matched site).

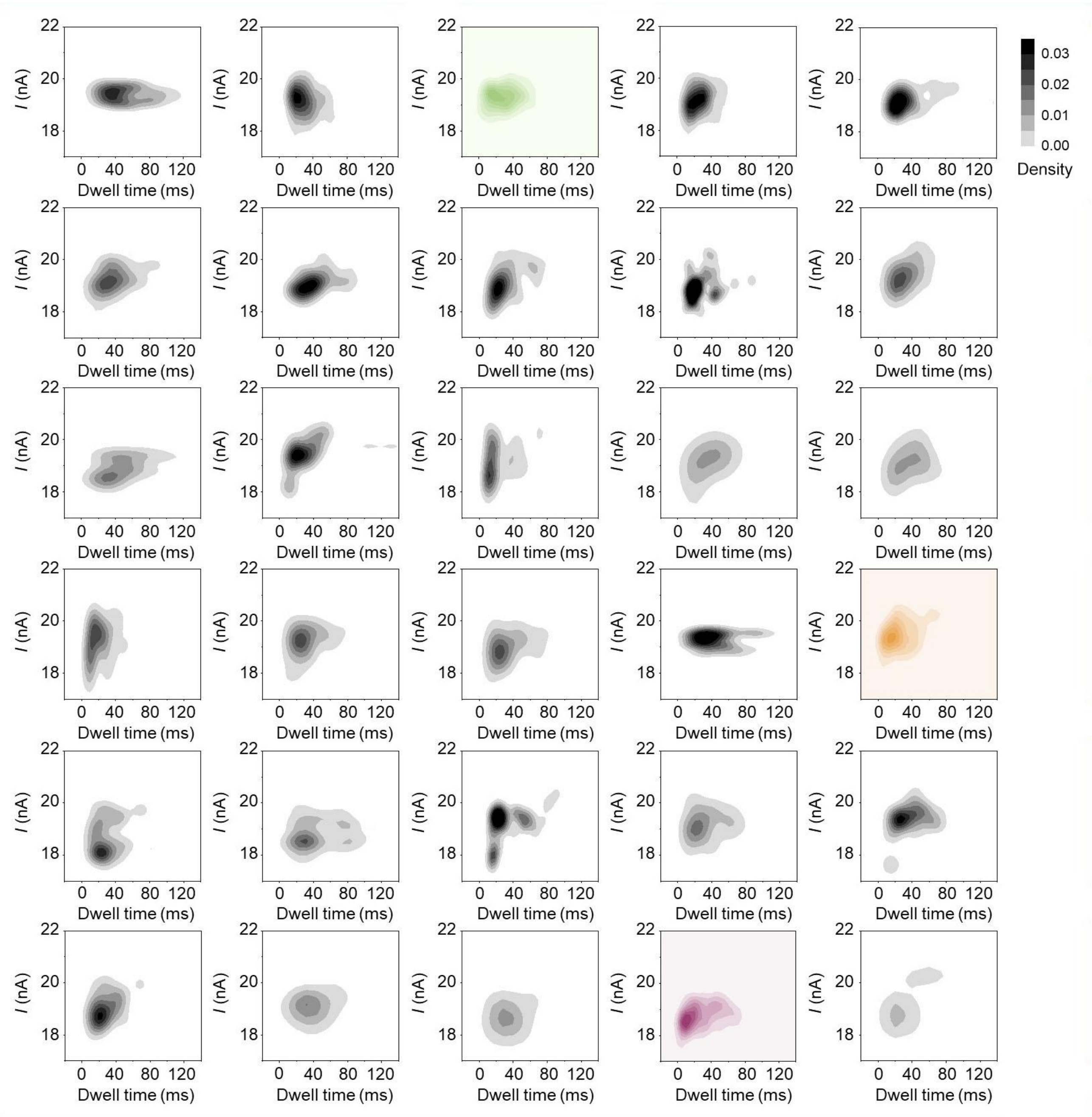

Supplementary Fig. 30 | Fingerprint map collection of 30 sites using a heterogeneous sequence (5'CGAUCUUCAUUGCCAAGCGGCUAGCUCAAA3'). Statical nucleotide information of heterogeneous sequence from upper left to bottom right: **5'**NNANNNNNNNNNNNNNNNNNU NNNNNNNNGN**3'** (A–Adenine; U–Uracil; C–Cytosine; G–Guanine; N–Non-matched site).

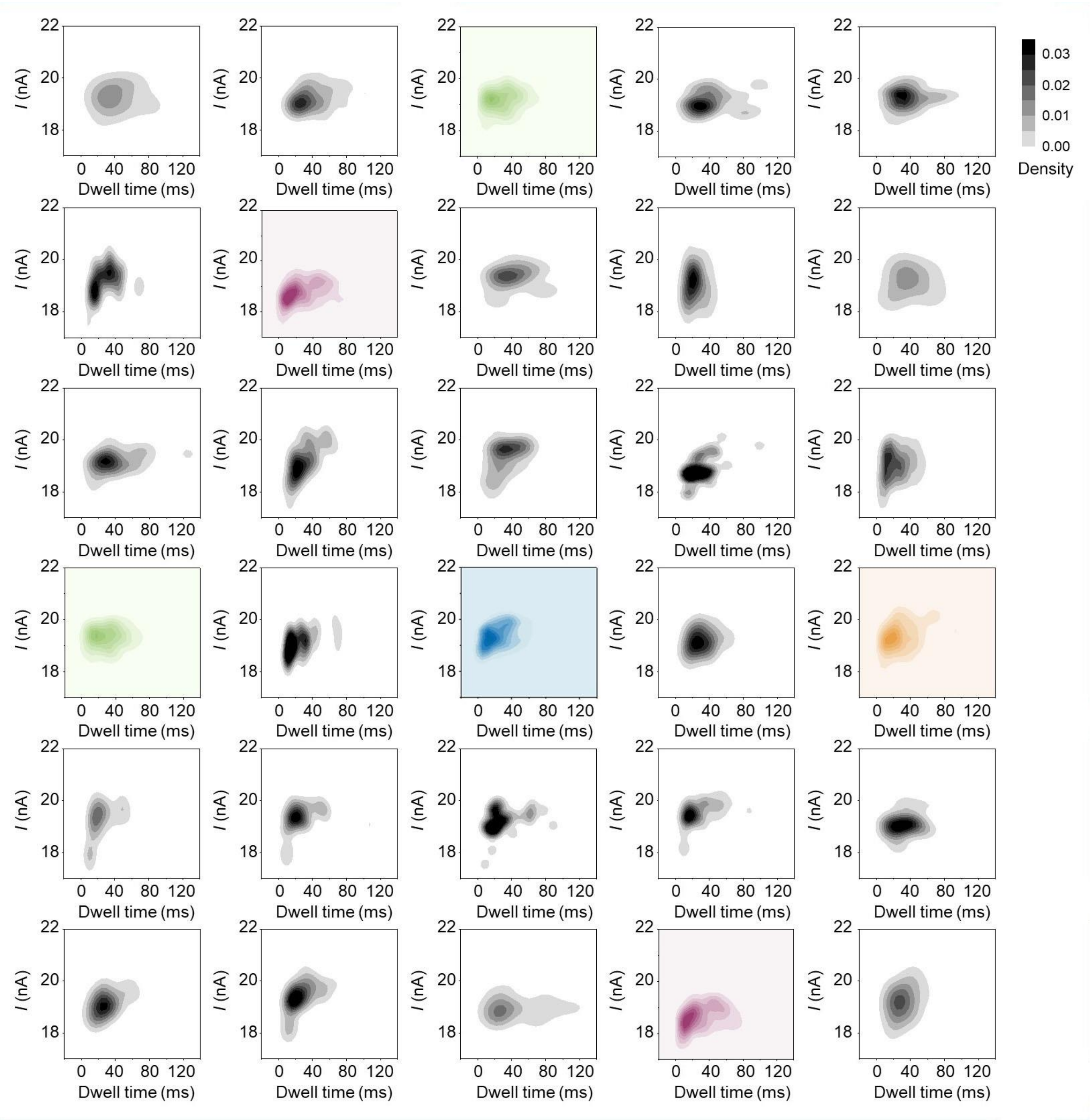

Supplementary Fig. 31 | Fingerprint map collection of 30 sites using a heterogeneous sequence (5'CGAUCUUCAUUGCCAAGCGGCUAGCUCAAA3'). Statical nucleotide information of heterogeneous sequence from upper left to bottom right: **5'**NNANNNGNNNNNNNNANCNUNNNNNNNNGN**3'** (A–Adenine; U–Uracil; C–Cytosine; G–Guanine; N–Non-matched site).

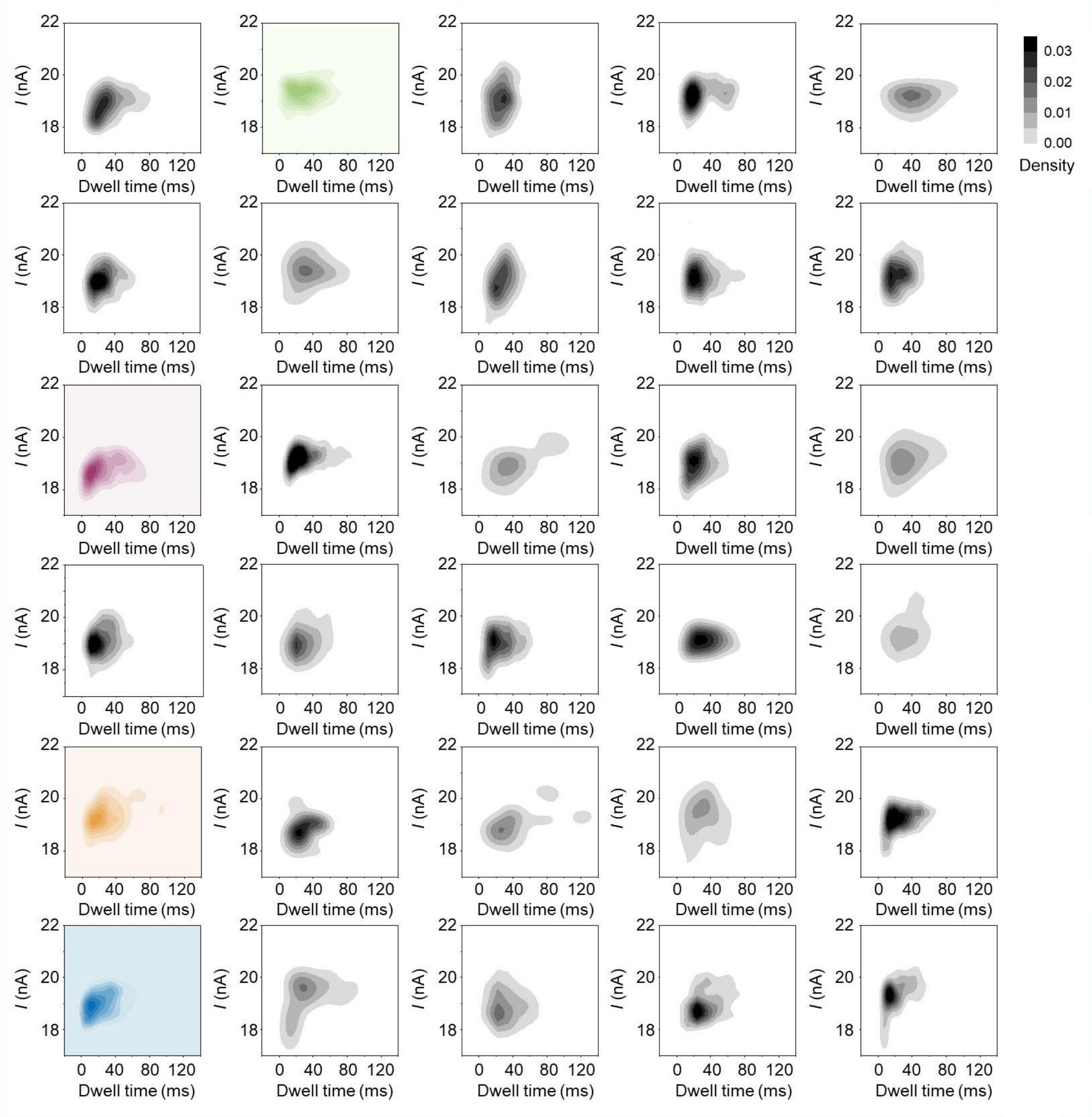

Supplementary Fig. 32 | Fingerprint map collection of 30 sites using a heterogeneous sequence (5'CGAUCUUCAUUGCCAAGCGGCUAGCUCAAA3'). Statical nucleotide information of heterogeneous sequence from upper left to bottom right: **5'**NANNNNNNNNGNNNNNNNNNUNNNNCNNNN**3'** (A–Adenine; U–Uracil; C–Cytosine; G–Guanine; N–Non-matched site).

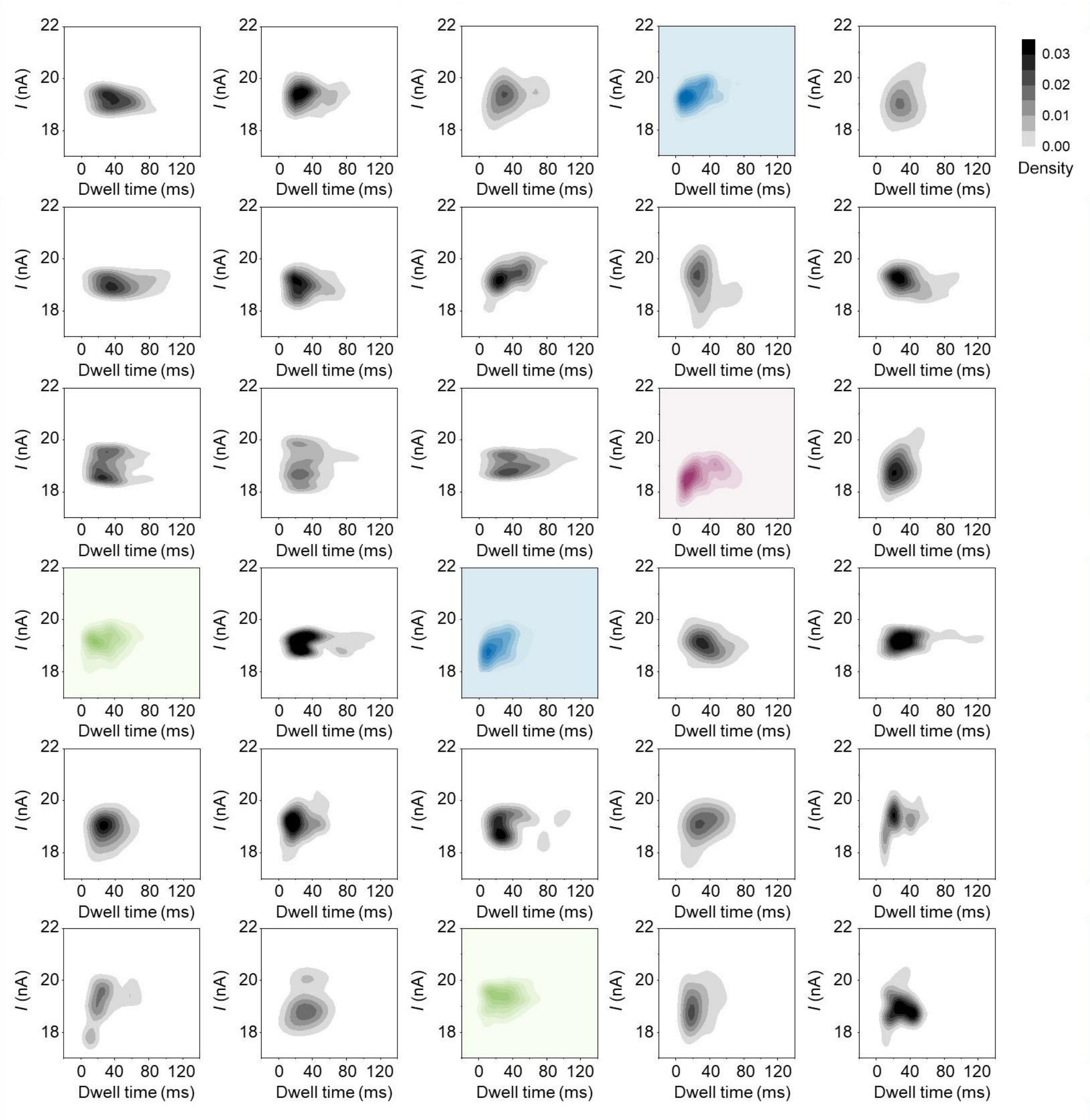

Supplementary Fig. 33 | Fingerprint map collection of 30 sites using a heterogeneous sequence (5’CGAUCUUCAUUGCCAAGCGGCUAGCUCAAA3’). Statical nucleotide information of heterogeneous sequence from upper left to bottom right: **5’**NNNCNNNNNNNNNNGNANCNNNNNNNNNANN**3’** (A–Adenine; U–Uracil; C–Cytosine; G–Guanine; N–Non-matched site).

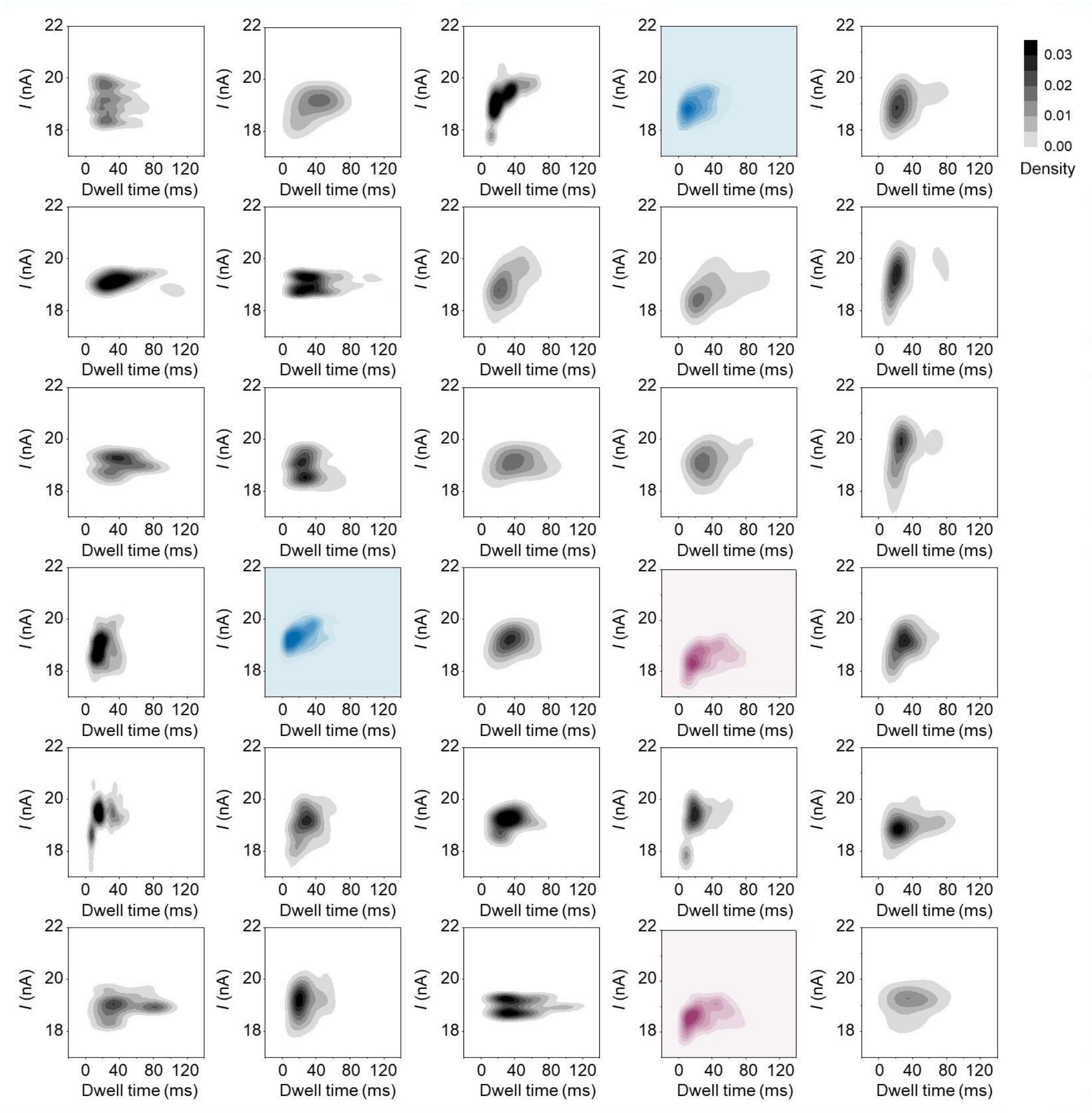

Supplementary Fig. 34 | Fingerprint map collection of 30 sites using a heterogeneous sequence (5'CGAUCUUCAUUGCCAAGCGGCUAGCUCAAA3'). Statical nucleotide information of heterogeneous sequence from upper left to bottom right: **5'**NNNCNNNNNNNNNNNNNCNGNNNNNNNNNGN**3'** (A–Adenine; U–Uracil; C–Cytosine; G–Guanine; N–Non-matched site).

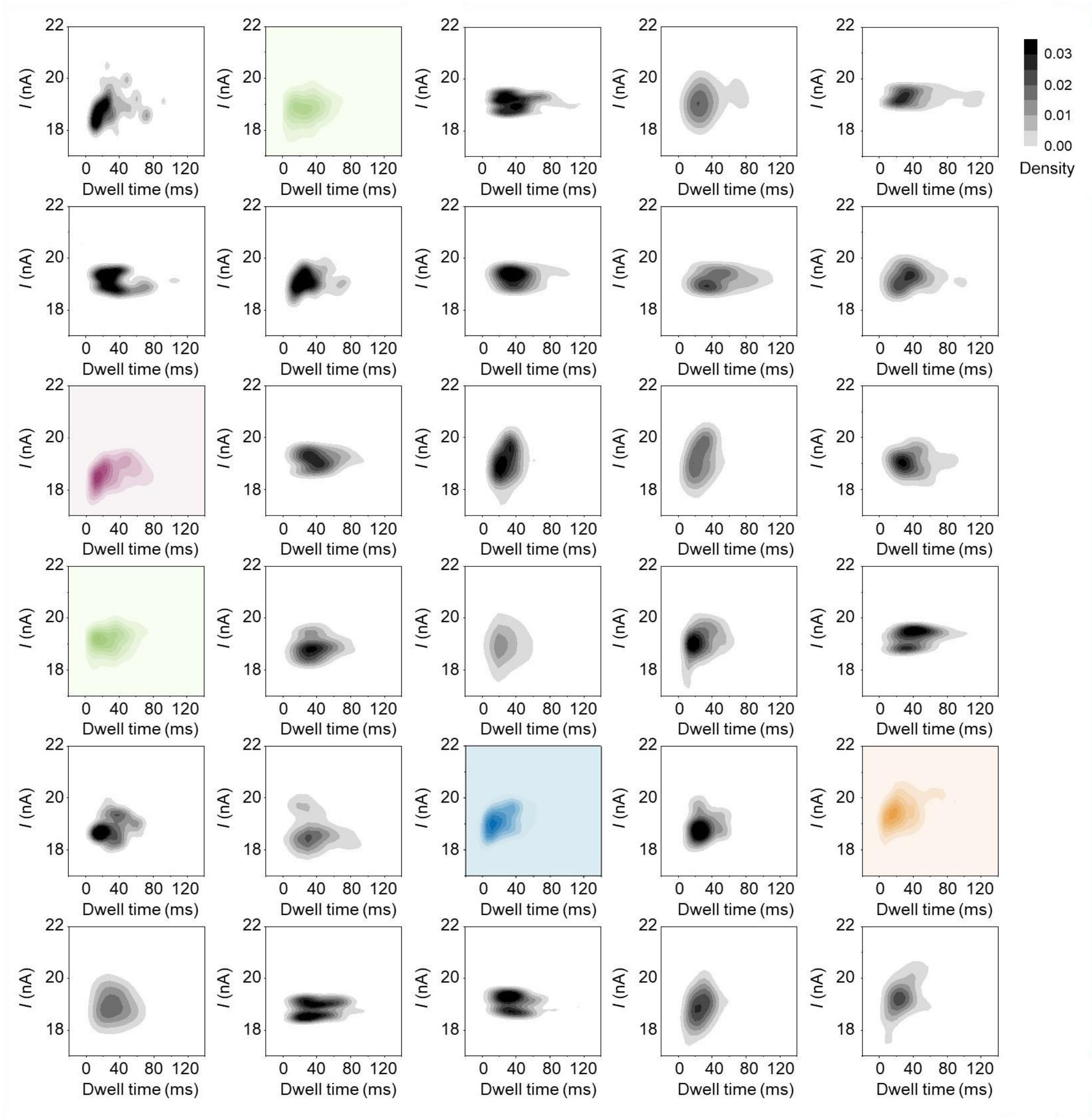

Supplementary Fig. 35 | Fingerprint map collection of 30 sites using a heterogeneous sequence (5'CGAUCUUCAUUGCCAAGCGGCUAGCUCAAA3'). Statical nucleotide information of heterogeneous sequence from upper left to bottom right: **5'**NANNNNNNNNGNNNNANNNNNNCNUNNNNN**3'** (A–Adenine; U–Uracil; C–Cytosine; G–Guanine; N–Non-matched site).

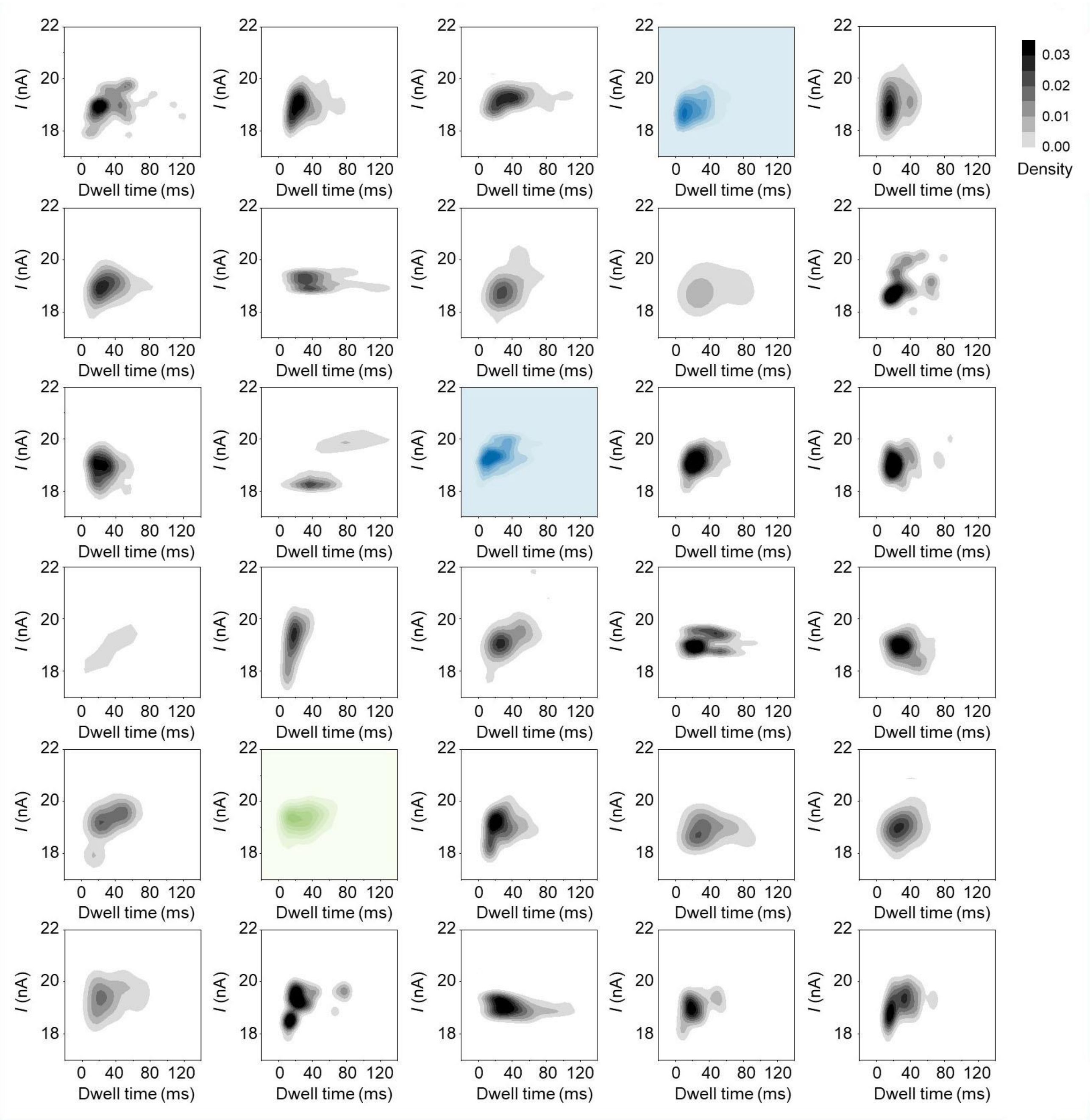

Supplementary Fig. 36 | Fingerprint map collection of 30 sites using a heterogeneous sequence (5'CGAUCUUCAUUGCCAAGCGGCUAGCUCAAA3'). Statical nucleotide information of heterogeneous sequence from upper left to bottom right: **5'**NNNCNNNNNNNNNCNNNNNNNNNANNNNNNNN**3'** (A–Adenine; U–Uracil; C–Cytosine; G–Guanine; N–Non-matched site).

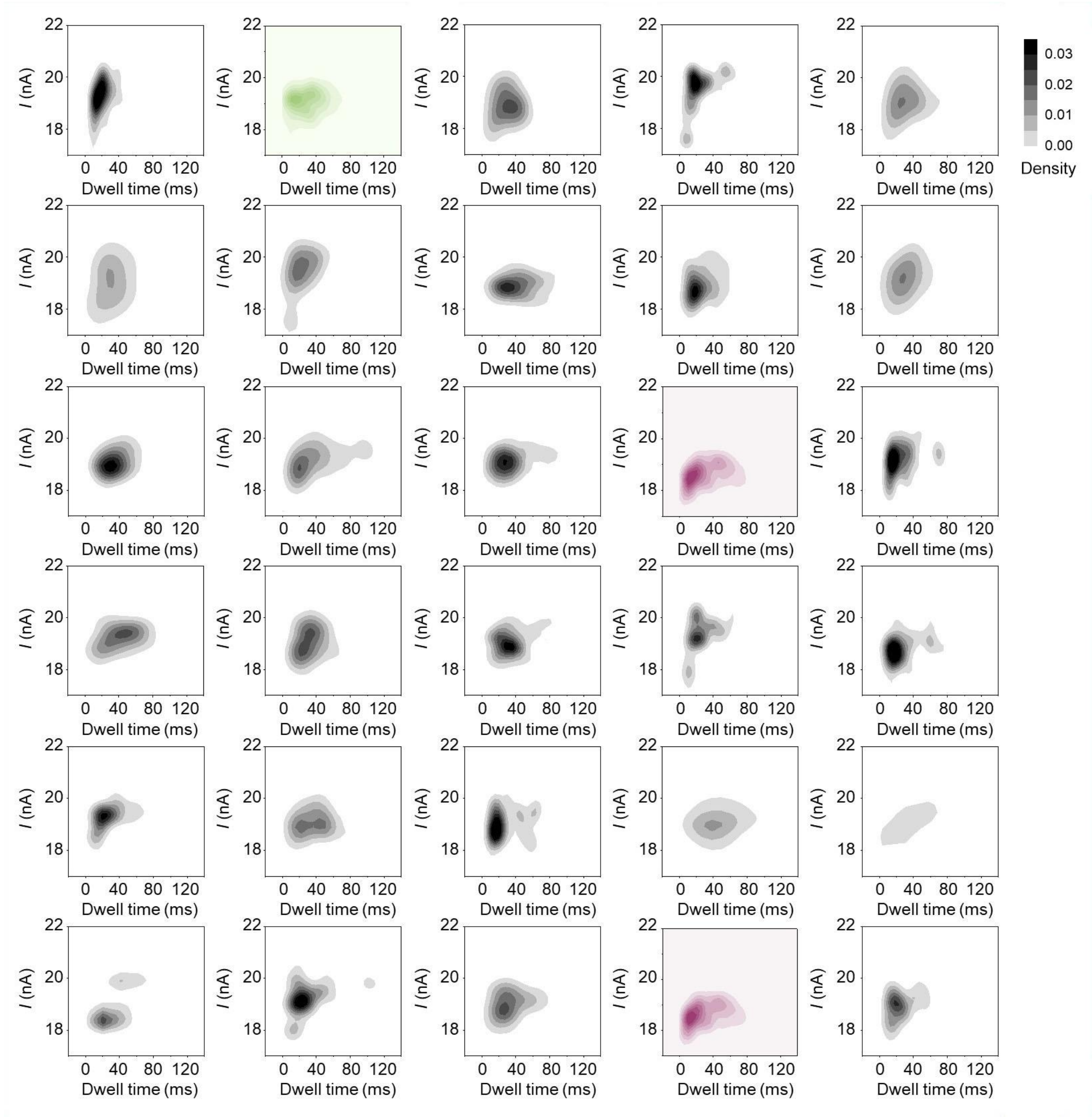

Supplementary Fig. 37 | Fingerprint map collection of 30 sites using a heterogeneous sequence (5’CGAUCUUCAUUGCCAAGCGGCUAGCUCAAA3’). Statical nucleotide information of heterogeneous sequence from upper left to bottom right: **5’**NANNNNNNNNNNNNGNNNNNNNNNNNNNNNGN**3’** (A–Adenine; U–Uracil; C–Cytosine; G–Guanine; N–Non-matched site).

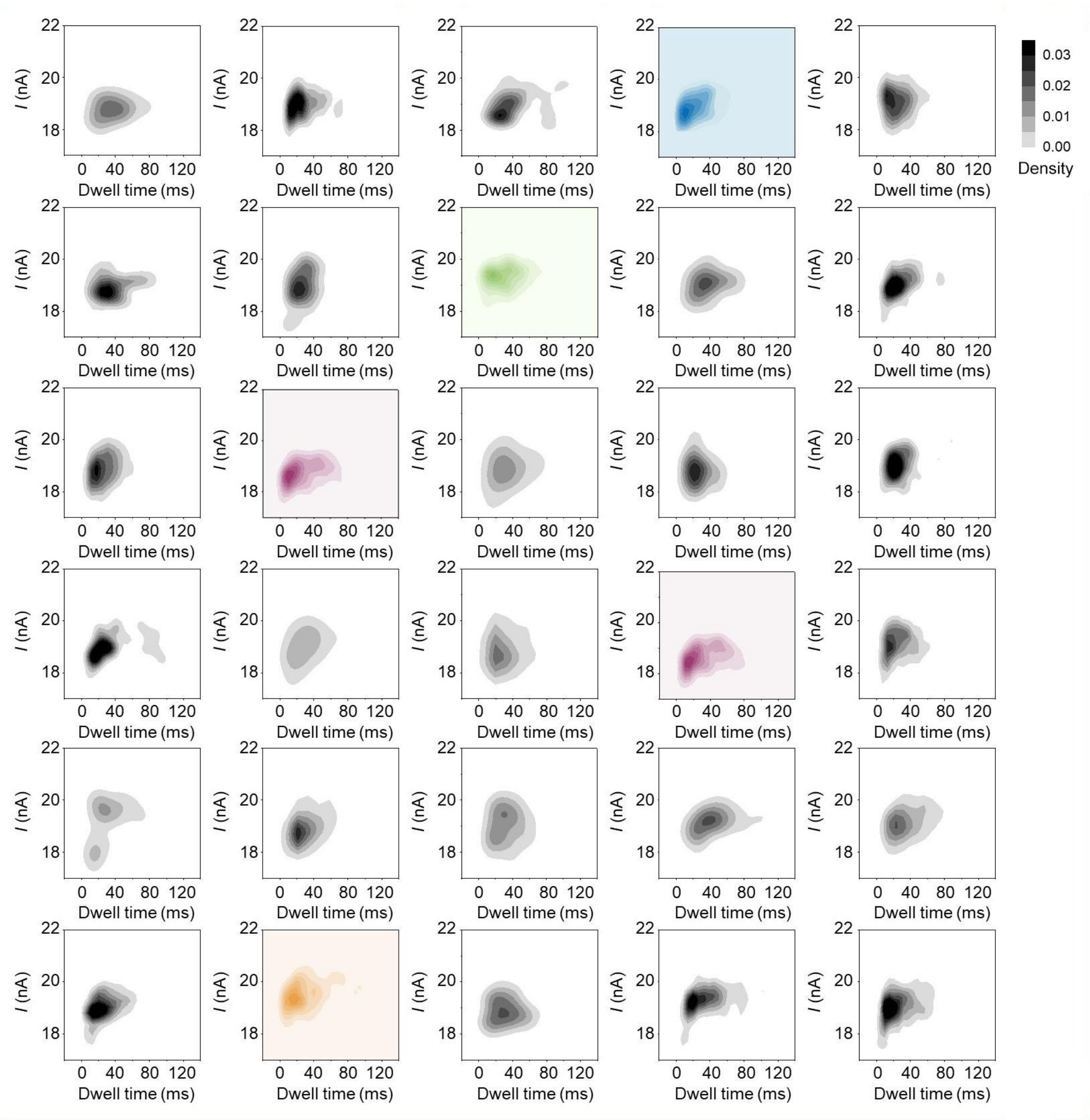

Supplementary Fig. 38 | Fingerprint map collection of 30 sites using a heterogeneous sequence (5'CGAUCUUCAUUGCCAAGCGGCUAGCUCAAA3'). Statical nucleotide information of heterogeneous sequence from upper left to bottom right: **5'**NNNCNNNANNNGNNNNNNNGNNNNNNNUNNN**3'** (A–Adenine; U–Uracil; C–Cytosine; G–Guanine; N–Non-matched site).

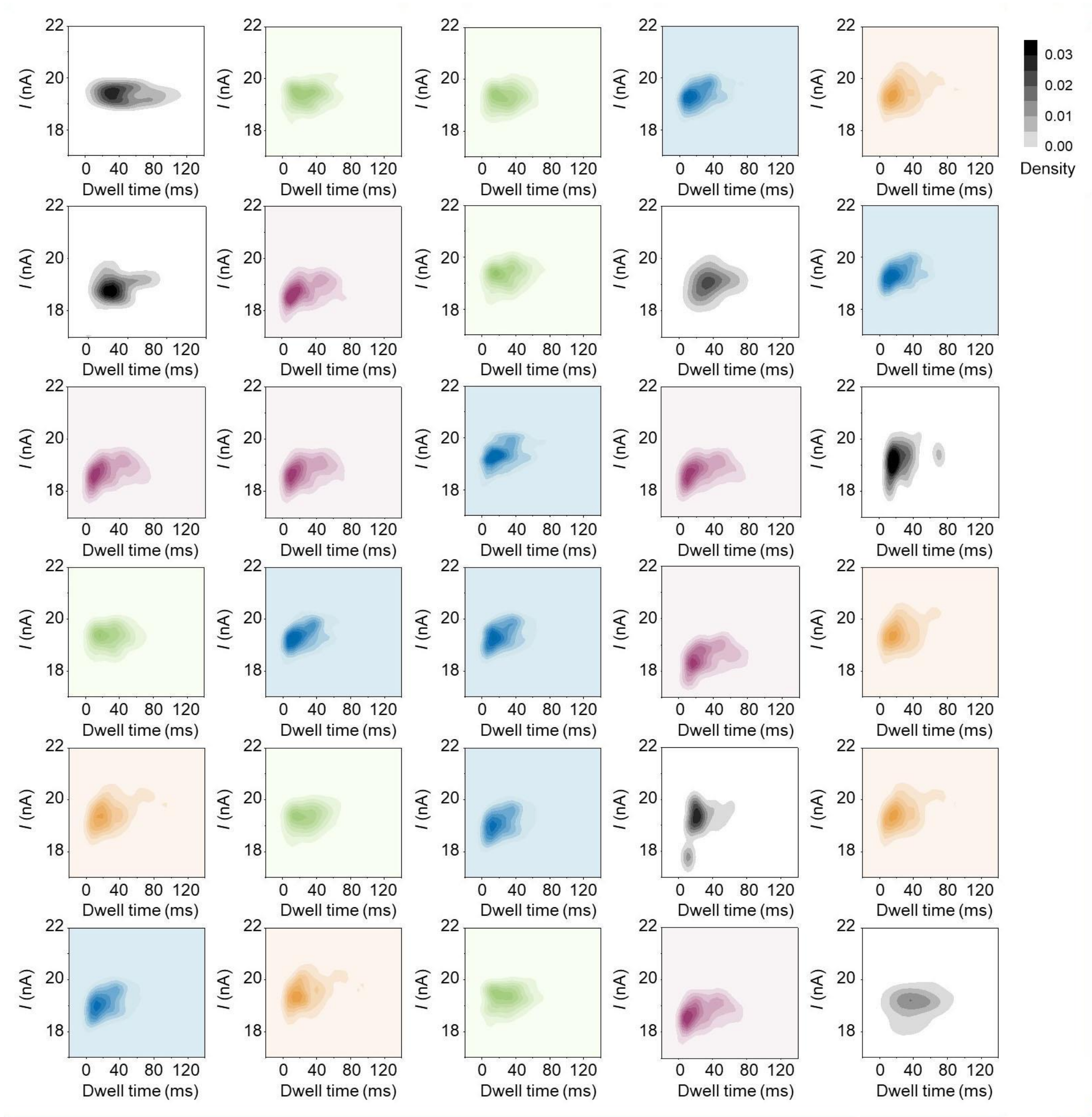

Supplementary Fig. 39 | Integrated fingerprint map collection of 30 sites using a heterogeneous sequence (5'CGAUCUUCAUUGCCAAGCGGCUAGCUCAAA3'). Statical nucleotide information of heterogeneous sequence from upper left to bottom right: **5'**NAACUNGANCGGCGNACCGU UACNUCUAGN**3'** (A–Adenine; U–Uracil; C–Cytosine; G–Guanine; N–Non-matched site).

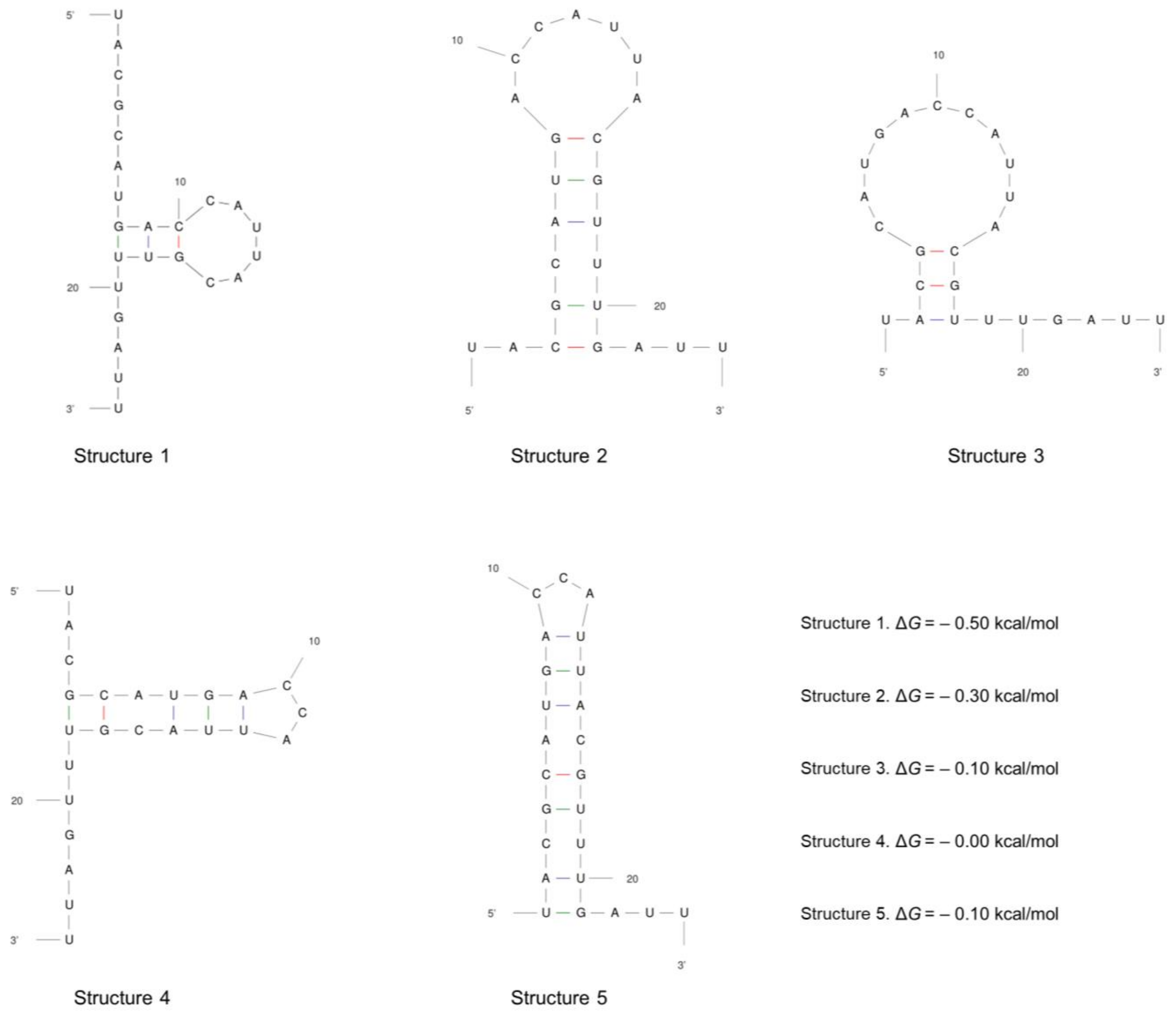

Supplementary Fig. 40 | Potential secondary structures of a *mccA* sequence. The pairing bond energy was ranked as red, purple, and green from high to low.

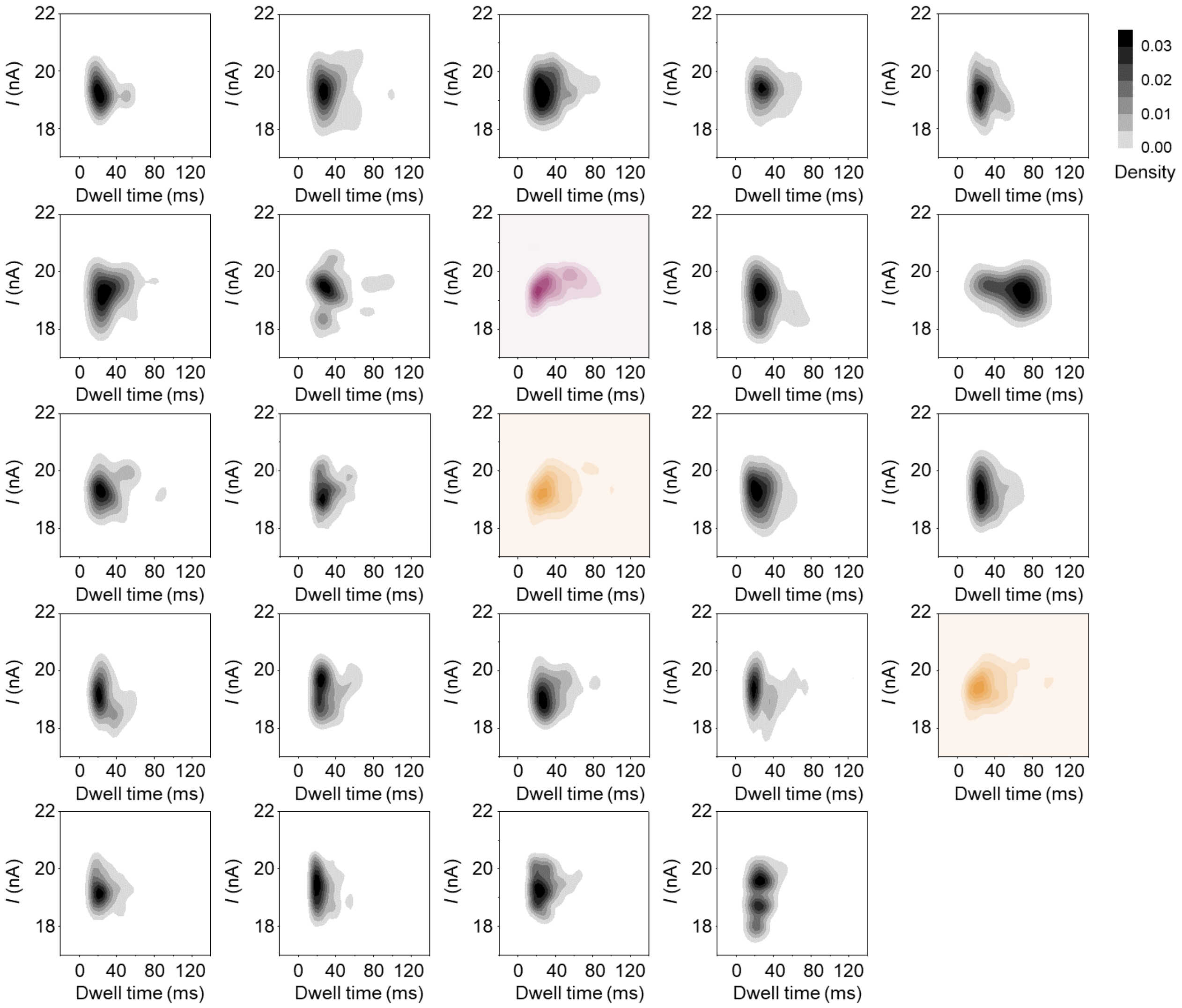

Supplementary Fig. 41 | Integrated fingerprint map collection of 24 sites for *mccA* gene (5' UACGCAU GACCAUUACGUUUGAUU3'). Statical nucleotide information of *mccA* gene sequence from upper left to bottom right: **5'**NNNNNNNGNNNNUNNNNNNUNNNN**3'** (A–Adenine; U–Uracil; C–Cytosine; G–Guanine; N–Non-matched site).

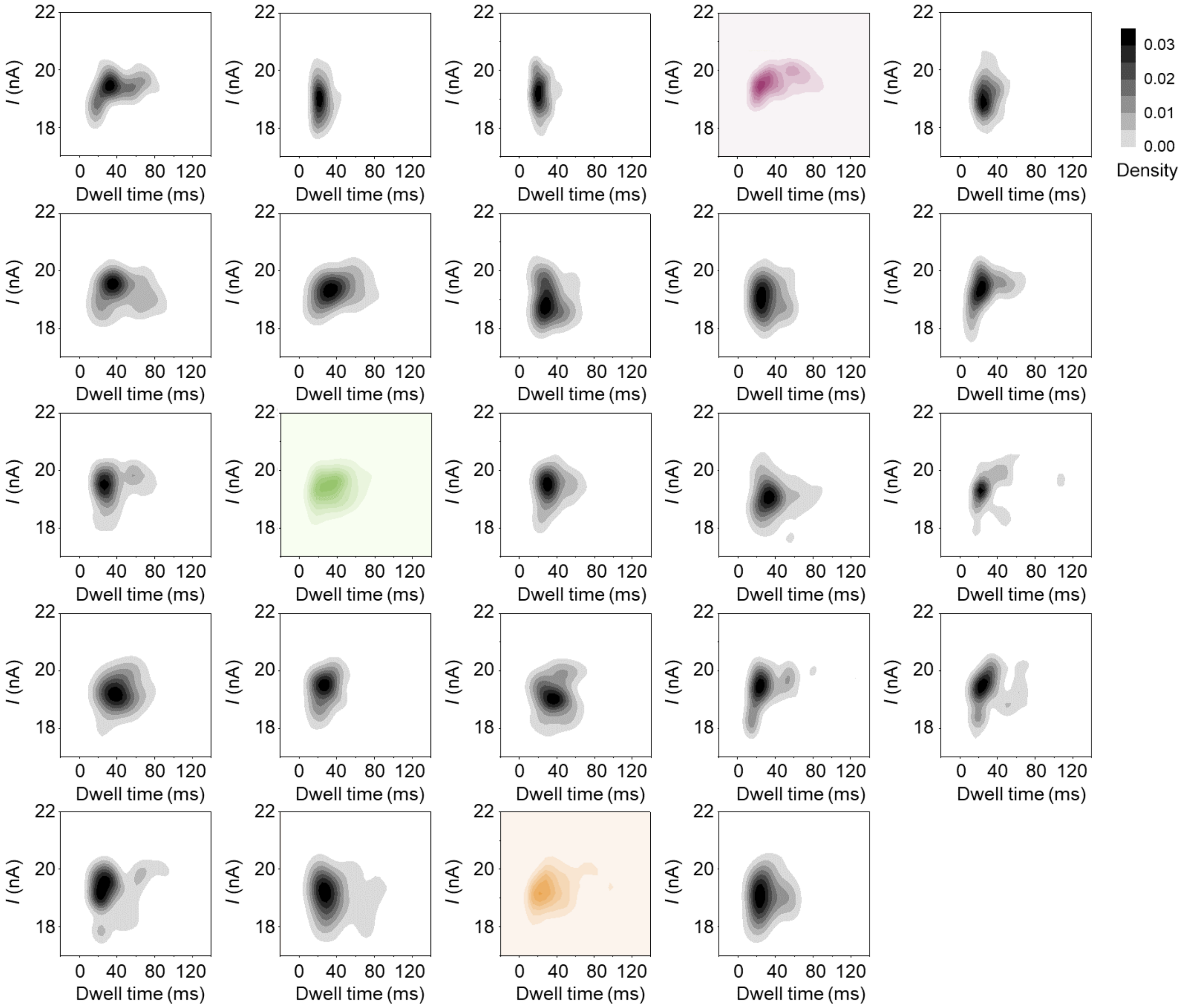

Supplementary Fig. 42 | Integrated fingerprint map collection of 24 sites for *mccA* gene (5' UACGCAU GACCAUUACGUUUGAUU3'). Statical nucleotide information of *mccA* gene sequence from upper left to bottom right: **5'**NNNGNNNNNNNANNNNNNNNNNUN**3'** (A–Adenine; U–Uracil; C–Cytosine; G–Guanine; N–Non-matched site).

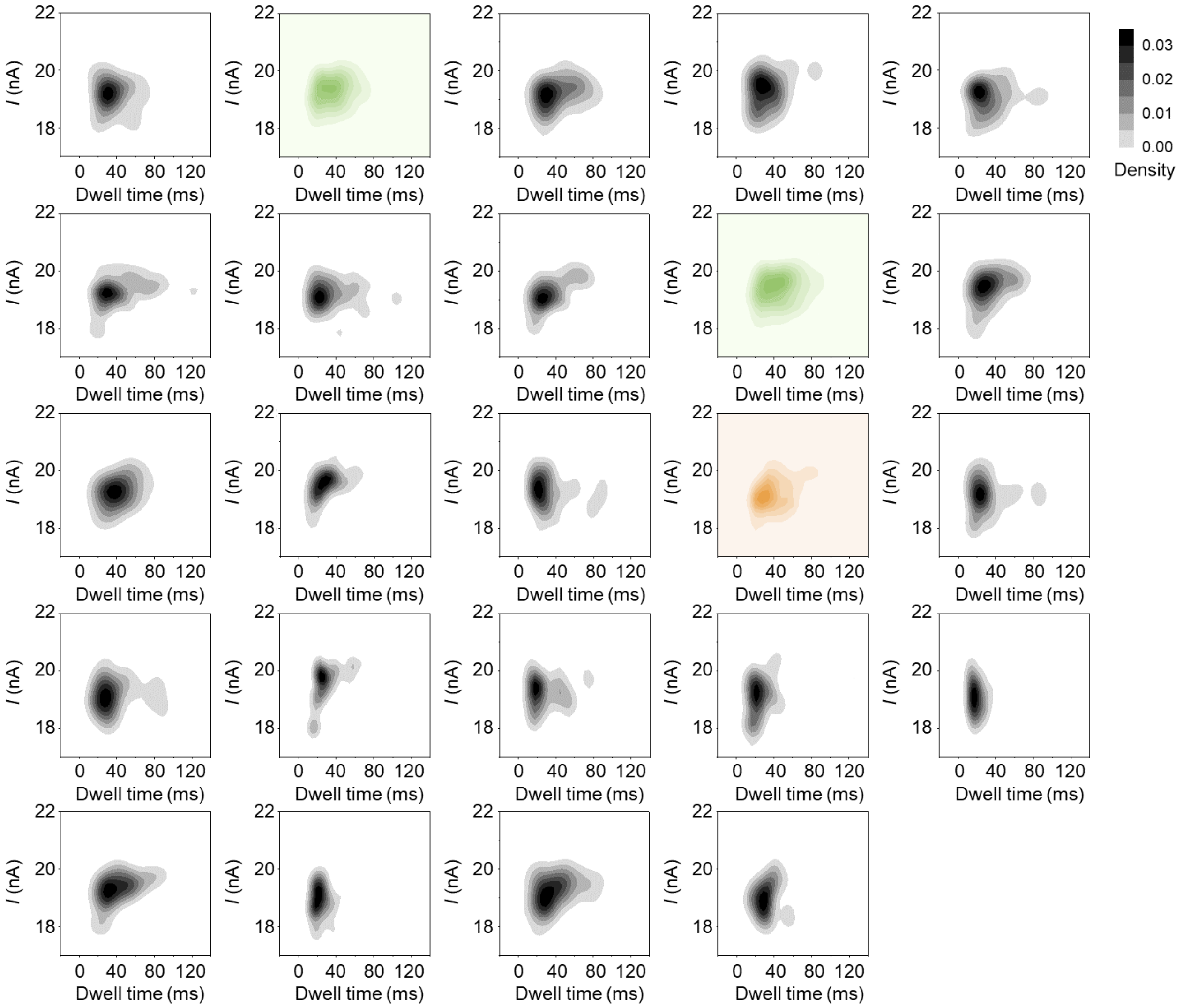

Supplementary Fig. 43 | Integrated fingerprint map collection of 24 sites for *mccA* gene (5' UACGCAU GACCAUUACGUUUGAUU3'). Statical nucleotide information of *mccA* gene sequence from upper left to bottom right: **5'**NANNNNNNANNNNUNNNNNNNNNN**3'** (A–Adenine; U–Uracil; C–Cytosine; G–Guanine; N–Non-matched site).

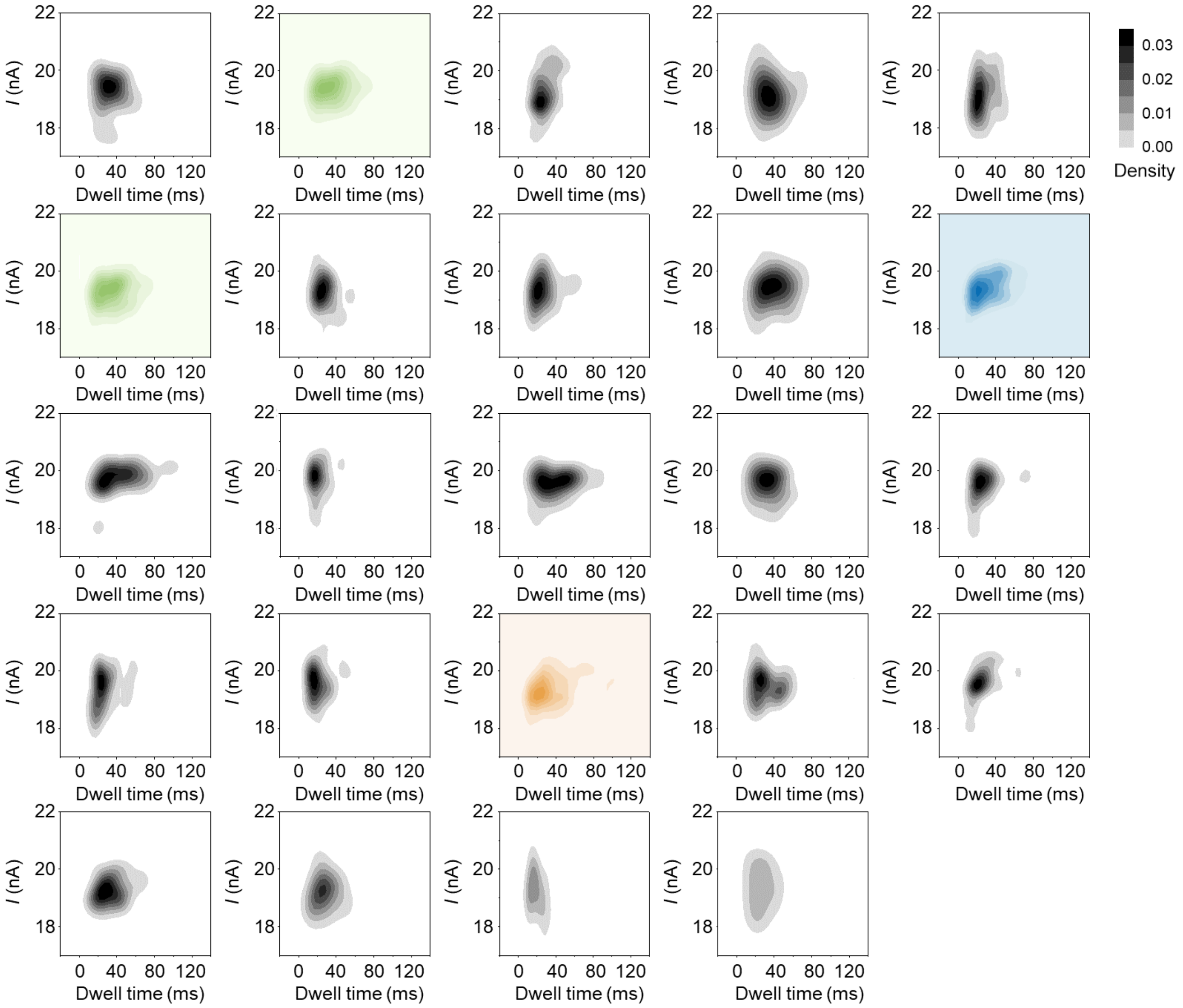

Supplementary Fig. 44 | Integrated fingerprint map collection of 24 sites for *mccA* gene (5' UACGCAU GACCAUUACGUUUGAUU3'). Statical nucleotide information of *mccA* gene sequence from upper left to bottom right: **5'**NANNNANNNCNNNNNNNNUNNNNNN**3'** (A–Adenine; U–Uracil; C–Cytosine; G–Guanine; N–Non-matched site).

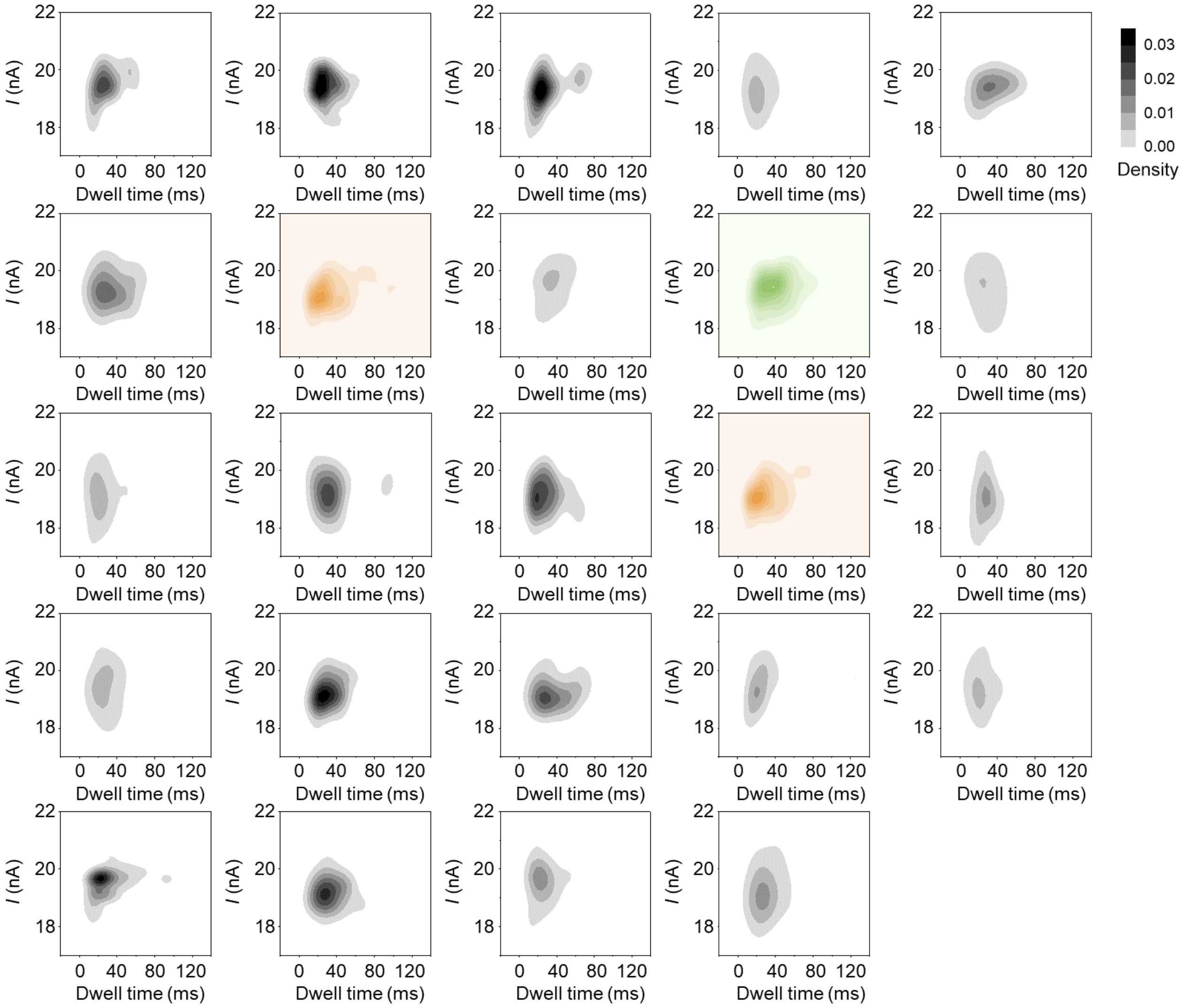

Supplementary Fig. 45 | Integrated fingerprint map collection of 24 sites for *mccA* gene (5' UACGCAU GACCAUUACGUUUGAUU3'). Statical nucleotide information of *mccA* gene sequence from upper left to bottom right: **5'**NNNNNNUNANNNNUNNNNNNNNNNN**3'** (A–Adenine; U–Uracil; C–Cytosine; G–Guanine; N–Non-matched site).

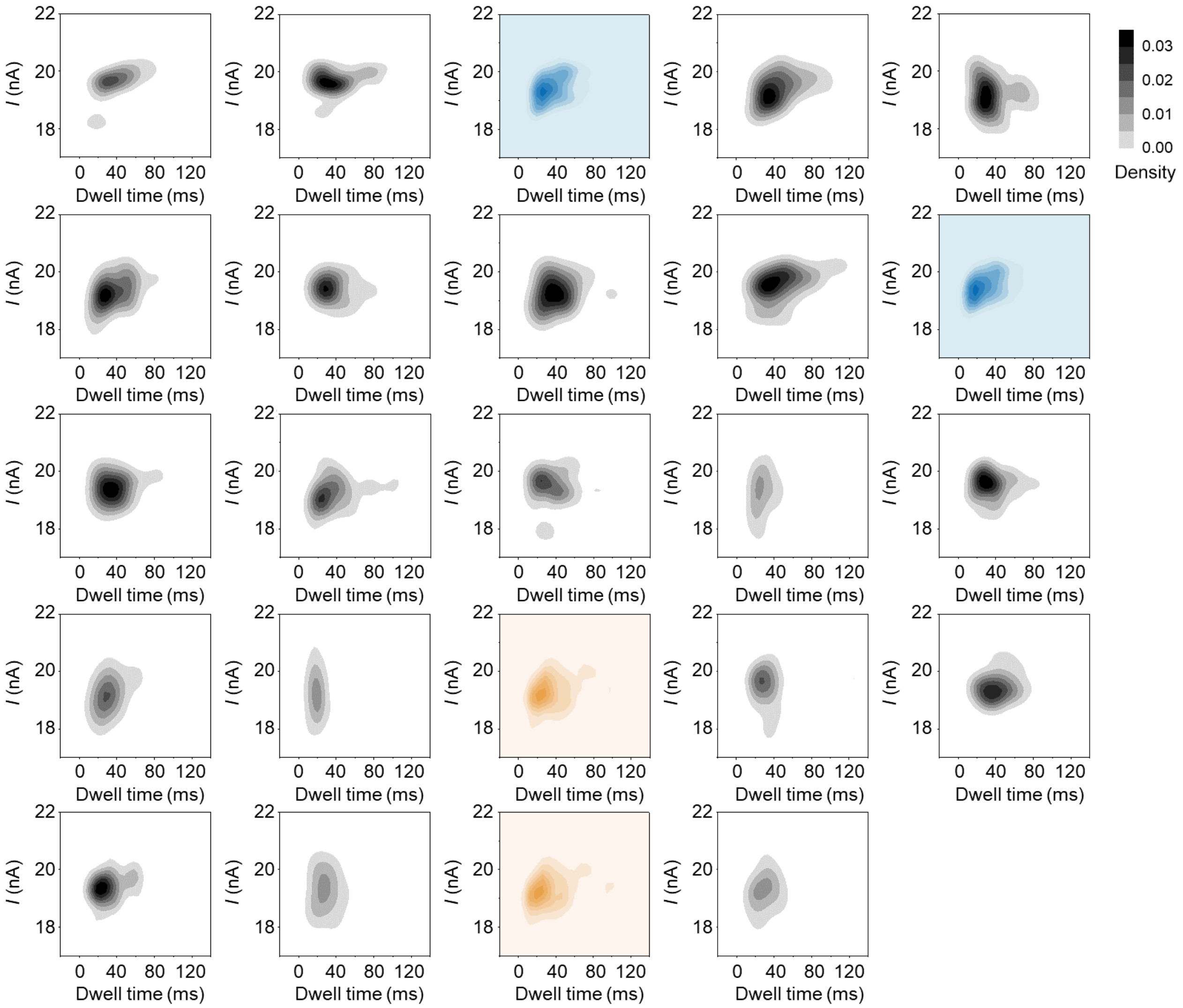

Supplementary Fig. 46 | Integrated fingerprint map collection of 24 sites for *mccA* gene (5' UACGCAU GACCAUUACGUUUGAUU3'). Statical nucleotide information of *mccA* gene sequence from upper left to bottom right: **5'**NNCNNNNNNCNNNNNNNUNNNNUN**3'** (A–Adenine; U–Uracil; C–Cytosine; G–Guanine; N–Non-matched site).

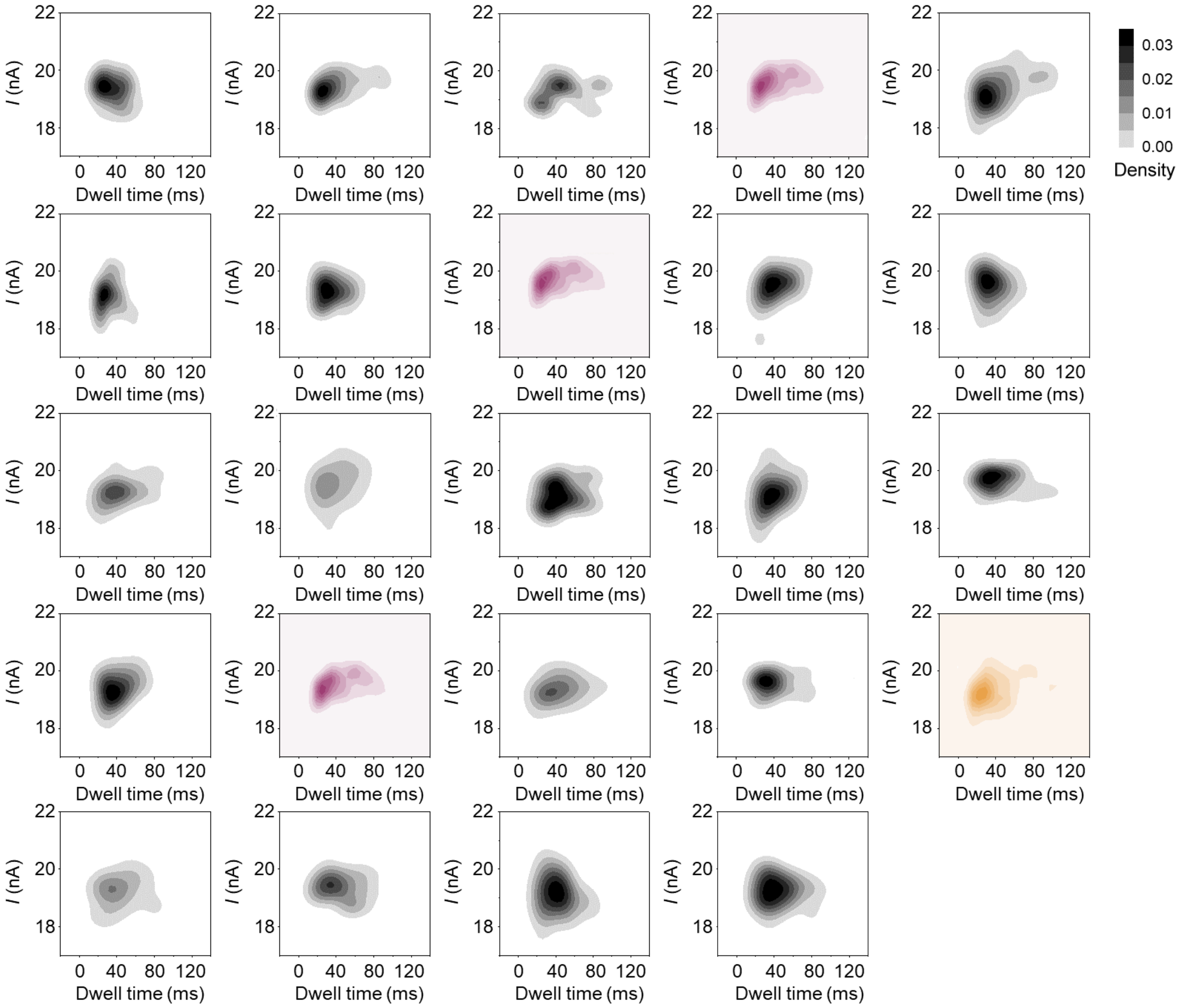

Supplementary Fig. 47 | Integrated fingerprint map collection of 24 sites for *mccA* gene (5' UACGCAU GACCAUUACGUUUGAUU3'). Statical nucleotide information of *mccA* gene sequence from upper left to bottom right: **5'**NNNGNNNGNNNNNNNNNGNNUNNNN**3'** (A–Adenine; U–Uracil; C–Cytosine; G–Guanine; N–Non-matched site).

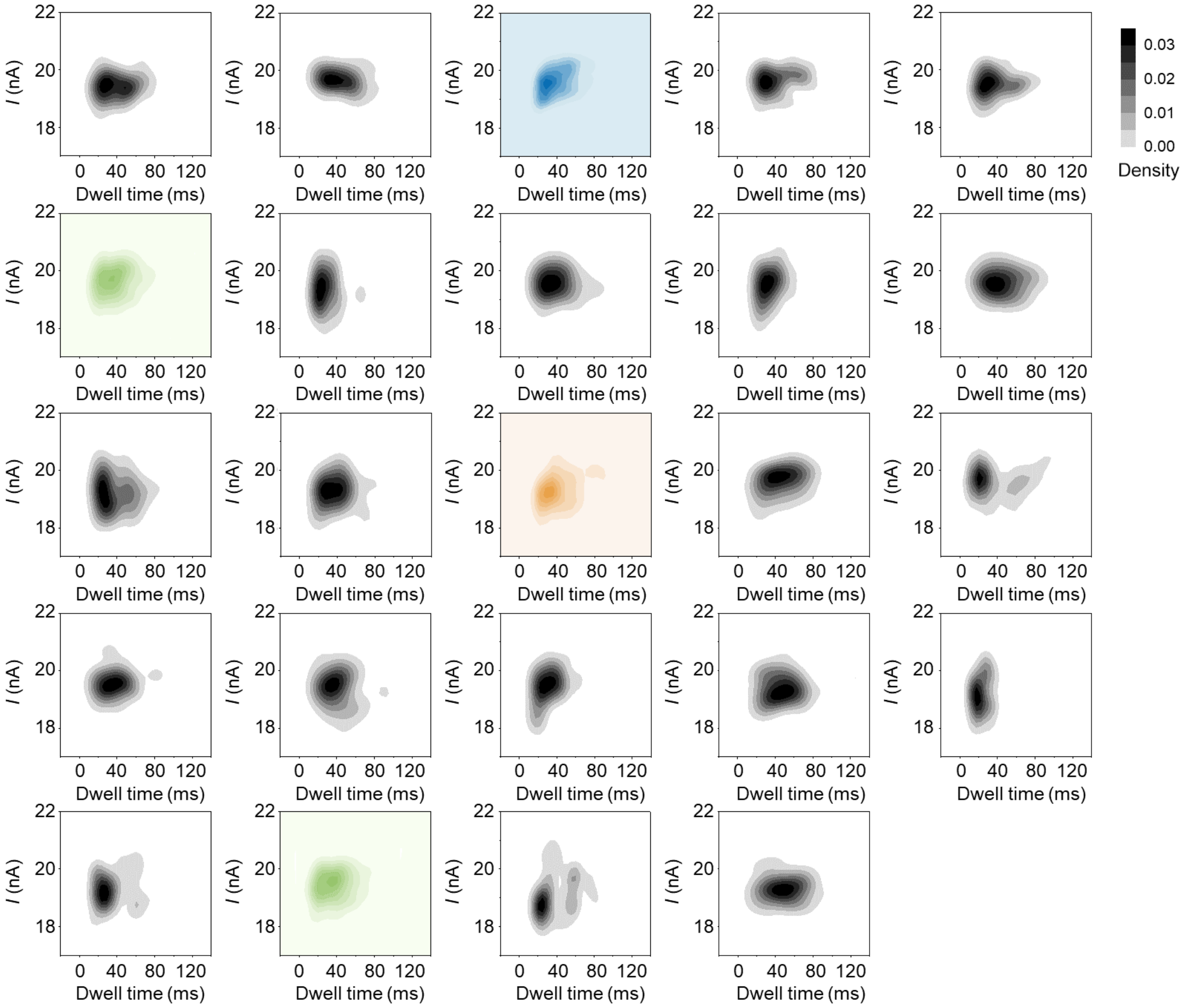

Supplementary Fig. 48 | Integrated fingerprint map collection of 24 sites for *mccA* gene (5' UACGCAU GACCAUUACGUUUGAUU3'). Statical nucleotide information of *mccA* gene sequence from upper left to bottom right: **5'**NNCNNANNNNNNUNNNNNNNNNANN**3'** (A–Adenine; U–Uracil; C–Cytosine; G–Guanine; N–Non-matched site).

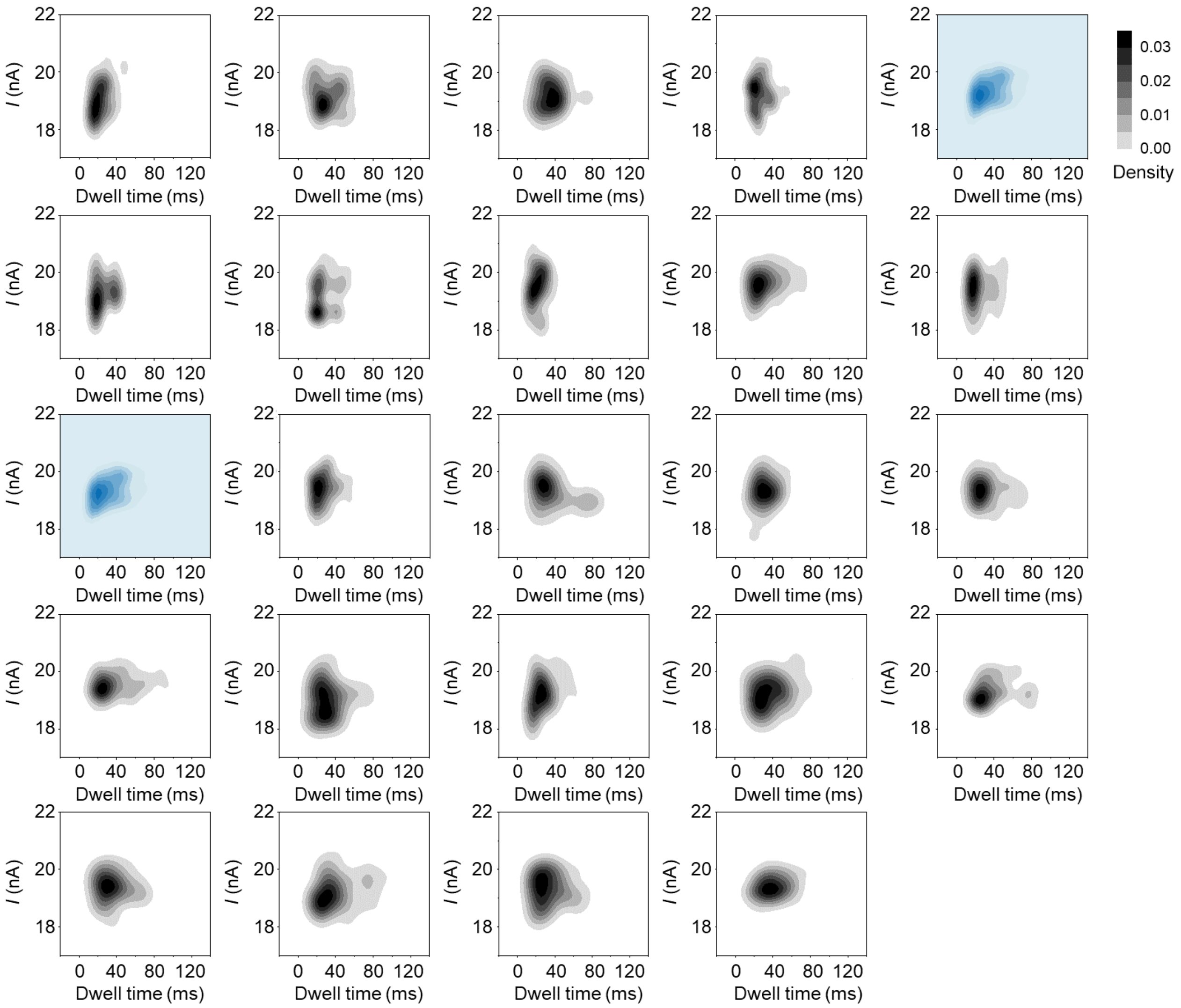

Supplementary Fig. 49 | Integrated fingerprint map collection of 24 sites for *mccA* gene (5' UACGCAU GACCAUUACGUUUGAUU3'). Statical nucleotide information of *mccA* gene sequence from upper left to bottom right: **5'**NNNNCNNNNNCNNNNNNNNNNNNNN**3'** (A–Adenine; U–Uracil; C–Cytosine; G–Guanine; N–Non-matched site).

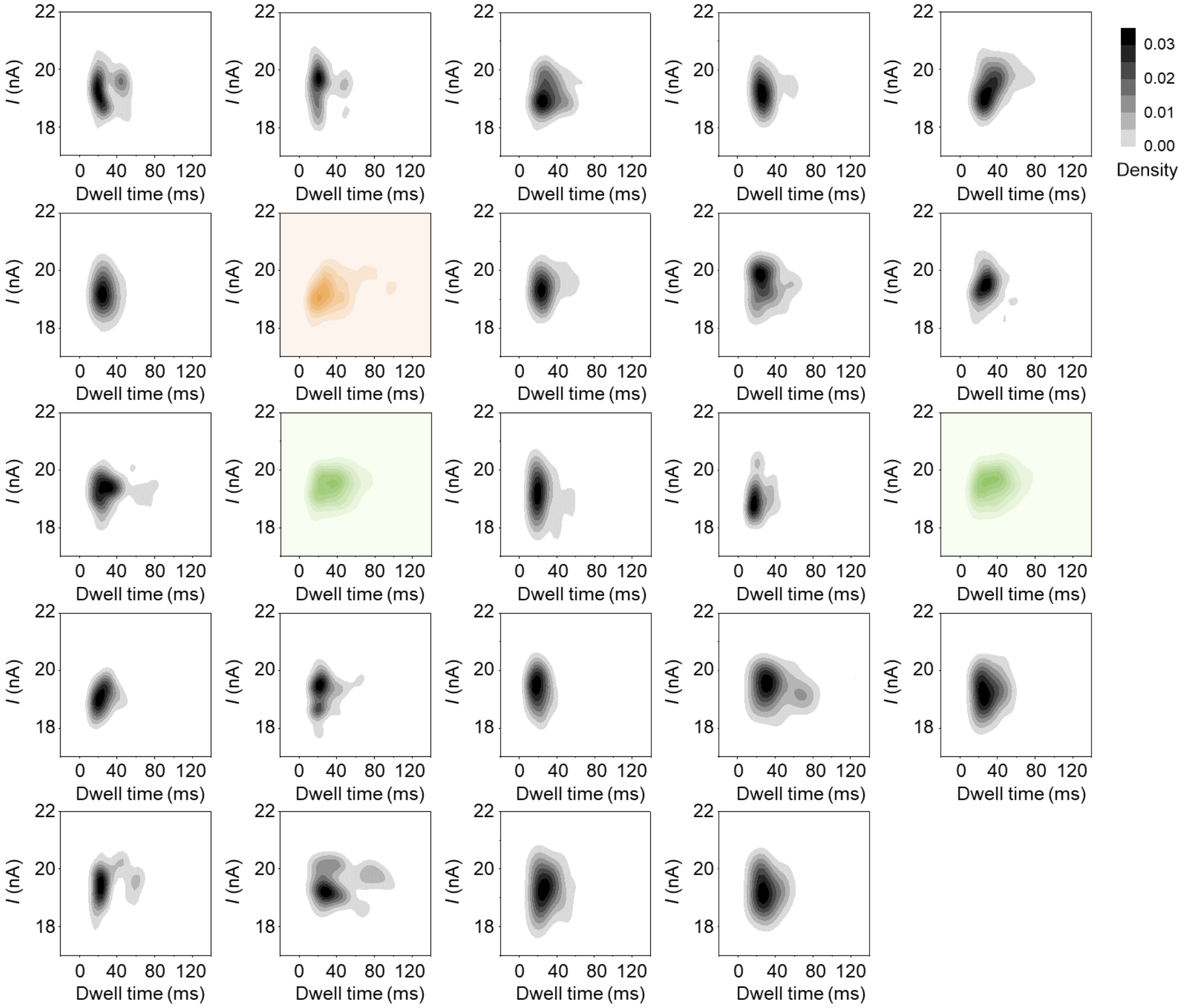

Supplementary Fig. 50 | Integrated fingerprint map collection of 24 sites for *mccA* gene (5' UACGCAU GACCAUUACGUUUGAUU3'). Statical nucleotide information of *mccA* gene sequence from upper left to bottom right: **5'**NNNNNNNUNNNNANNANNNNNNNNNN**3'** (A–Adenine; U–Uracil; C–Cytosine; G–Guanine; N–Non-matched site).

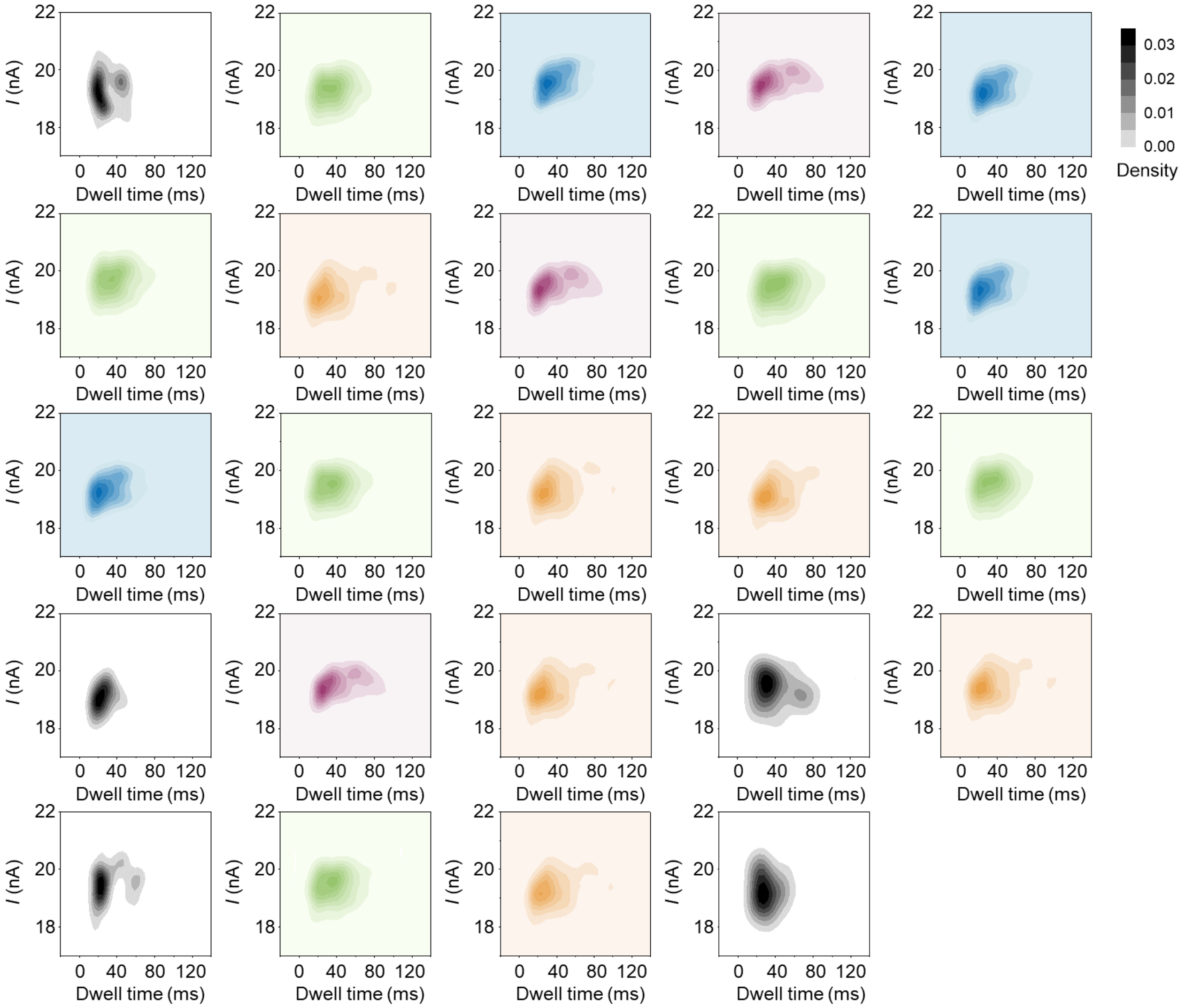

Supplementary Fig. 51 | Integrated fingerprint map collection of 24 sites for *mccA* gene (5' UACGCAU GACCAUUACGUUUGAUU3'). Statical nucleotide information of *mccA* gene sequence from upper left to bottom right: **5'**NACGCAUGACCAUUANGUNUNAUN**3'** (A–Adenine; U–Uracil; C–Cytosine; G–Guanine; N–Non-matched site).

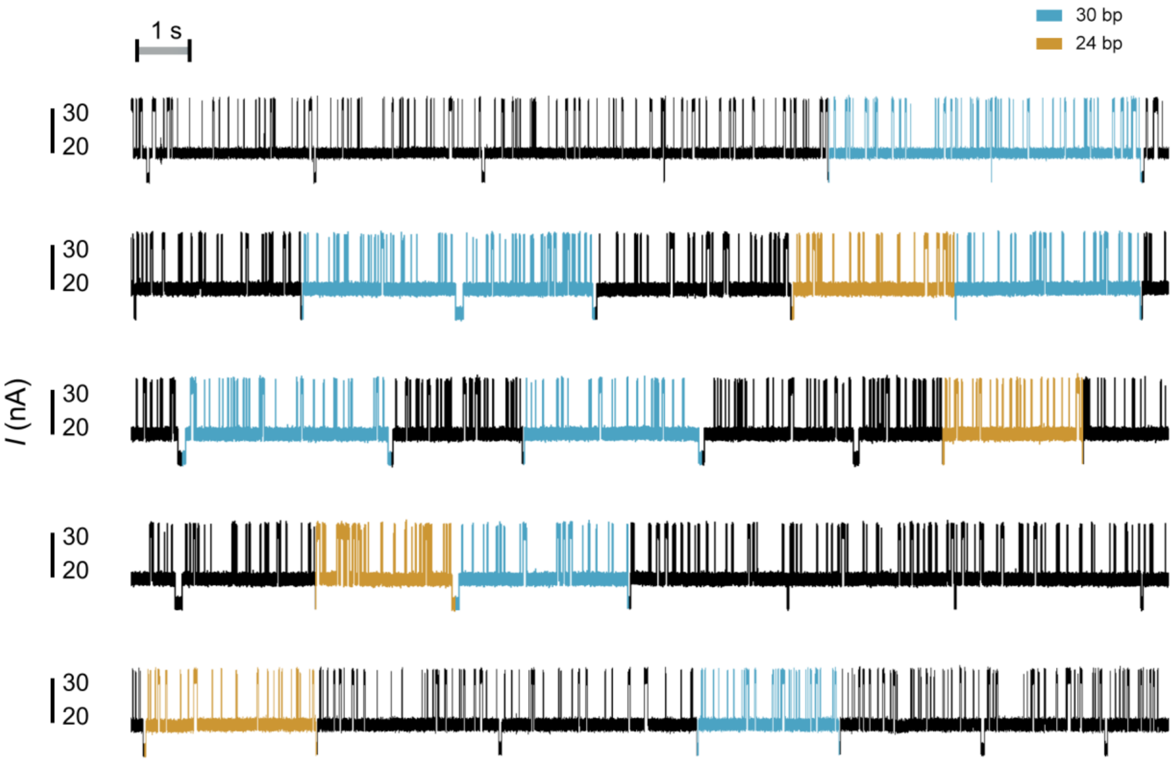

Supplementary Fig. 52 | Five real–time electrical trajectories (each trace duration: 20 s) of the degradation process of the mixed sample consisting of an artificially designed sequence (30 bp in length) and a *mccA* gene sequence (24 bp in length). Temperature: 37 °C, RNA substrate: 0.5 mmol•L^{-1} (artificially designed sequence), RNA substrate: 0.5 mmol•L^{-1} (*mccA* gene sequence), $MgCl_2$: 2 mmol•L^{-1} and H_3PO_4: 8 mM•L^{-1}.

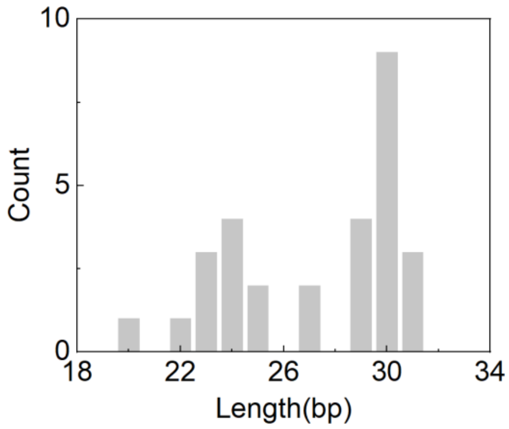

Supplementary Fig. 53 | The statistical distribution of the sequence length for five real–time electrical trajectories from the measurements of the mixed sample.

Supplementary Note 8. The assay data of the regulation on the Debye length in the buffer solution.

Supplementary Table 2 | A table of the assay data about the regulation of the Debye length.

Tris-HCl pH=7.6	λ_D (nm)	ionic strength (mM)
×1	1.13	59.30
×0.1	3.58	5.93
×0.01	11.32	0.59
