# Supplementary material for: Single-exonuclease nanocircuits reveal the RNA degradation dynamics of PNPase and demonstrate potential for RNA sequencing
Source: Nat Commun. 2023 Feb 1;14:552. doi: 10.1038/s41467-023-36278-6 (PMC9892577; doi:10.1038/s41467-023-36278-6)
Supplement: Supplementary file 3 — Reporting Summary [file 41467_2023_36278_MOESM3_ESM.pdf]

nature portfolio

Corresponding author(s): Lidong Li and Xuefeng Guo

Last updated by author(s): Jan 16, 2023

Reporting Summary

Nature Portfolio wishes to improve the reproducibility of the work that we publish. This form provides structure for consistency and transparency in reporting. For further information on Nature Portfolio policies, see our Editorial Policies and the Editorial Policy Checklist.

Statistics

For all statistical analyses, confirm that the following items are present in the figure legend, table legend, main text, or Methods section.

n/a	Confirmed	
	x	The exact sample size (n) for each experimental group/condition, given as a discrete number and unit of measurement
	x	A statement on whether measurements were taken from distinct samples or whether the same sample was measured repeatedly
x		The statistical test(s) used AND whether they are one- or two-sided<br>*Only common tests should be described solely by name; describe more complex techniques in the Methods section.*
	x	A description of all covariates tested
	x	A description of any assumptions or corrections, such as tests of normality and adjustment for multiple comparisons
	x	A full description of the statistical parameters including central tendency (e.g. means) or other basic estimates (e.g. regression coefficient) AND variation (e.g. standard deviation) or associated estimates of uncertainty (e.g. confidence intervals)
x		For null hypothesis testing, the test statistic (e.g. F, t, r) with confidence intervals, effect sizes, degrees of freedom and P value noted<br>*Give P values as exact values whenever suitable.*
x		For Bayesian analysis, information on the choice of priors and Markov chain Monte Carlo settings
	x	For hierarchical and complex designs, identification of the appropriate level for tests and full reporting of outcomes
x		Estimates of effect sizes (e.g. Cohen's d, Pearson's r), indicating how they were calculated

Our web collection on statistics for biologists contains articles on many of the points above.

Software and code

Policy information about availability of computer code

Data collection	The I–t curve were sampled by the HF2LI lock-in amplifier at 300 mV bias, 5 kHz bandwidth. The current signal was amplified by a DL1211 amplifier and then recorded by a high-speed acquisition card from NIDAQ at a rate of 57,600 samples per second.
Data analysis	The I–t curves can be analysed by the QuB 2.0.0.32 software. The raw data can be opened by matlab 2019a. The data statistics can be handled with origin 2019b.

For manuscripts utilizing custom algorithms or software that are central to the research but not yet described in published literature, software must be made available to editors and reviewers. We strongly encourage code deposition in a community repository (e.g. GitHub). See the Nature Portfolio guidelines for submitting code & software for further information.

Data

Policy information about availability of data

All manuscripts must include a data availability statement. This statement should provide the following information, where applicable:

- Accession codes, unique identifiers, or web links for publicly available datasets
- A description of any restrictions on data availability
- For clinical datasets or third party data, please ensure that the statement adheres to our policy

All the databases/datasets used in the study along with appropriately accessible links/accession-codes in the manuscript under the “Data availability” section as well as in this reporting summary. https://zenodo.org/record/7533205.

Human research participants

Policy information about studies involving human research participants and Sex and Gender in Research.

Reporting on sex and gender	This study does not involve human research participants.
Population characteristics	See above.
Recruitment	Not applicable.
Ethics oversight	Not applicable.

Note that full information on the approval of the study protocol must also be provided in the manuscript.

Field-specific reporting

Please select the one below that is the best fit for your research. If you are not sure, read the appropriate sections before making your selection.

☐ Life sciences ☐ Behavioural & social sciences ☒ Ecological, evolutionary & environmental sciences

For a reference copy of the document with all sections, see nature.com/documents/nr-reporting-summary-flat.pdf

Ecological, evolutionary & environmental sciences study design

All studies must disclose on these points even when the disclosure is negative.

Study description	We established a point-functionalized silicon nanowire as a biosensor for real-time sampling of RNA degradation dynamics at single-molecule resolution.
Research sample	A single intrinsically PNPase obtained from E.coli cell was covalently conjugated to the silicon nanowire through chemical engineering, and its conformational transition dynamics was characterized as current fluctuations. PNPase was chosen for the research by the trait that it has the step-by- step degradation capacity and appropriate time-scale compatibility.
Sampling strategy	An in-situ real-time electrical single-molecule detection which has the capability of single-event tracking. Sampling apparatus equipped with 17us temporal resolution enable us recording the detailed dynamic behavior of a single PNPase (millisecond spatial scale) in 20 s.
Data collection	The current signal of the SiNW loop was amplified by a DL1211 amplifier and collected by Zurich instruments to the hard disk of the computer by Zhiheng Yang.
Timing and spatial scale	The current signal recorded by a high-speed acquisition card from NIDAQ at a rate of 57,600 samples per second, which can record the detailed biological behavior of a single PNPase in 20 s. The samples were detected in 1-2 days, considering the biologically active property.
Data exclusions	No data are excluded from the analyses.
Reproducibility	Although values of the current levels varied from read to read among different SiNW-FET devices, the current conversion patterns and the dependence of the testing conditions were highly reproducible. The data was collected from three different devices to confirm the reproducibility.
Randomization	The samples were obtained with ultra-high purity to avoid the experimental randomization error.
Blinding	The experiments were proceeded by all the members in our team without blinding operation.

Did the study involve field work? ☐ Yes ☒ No

Reporting for specific materials, systems and methods

We require information from authors about some types of materials, experimental systems and methods used in many studies. Here, indicate whether each material, system or method listed is relevant to your study. If you are not sure if a list item applies to your research, read the appropriate section before selecting a response.

Materials & experimental systems

n/a	Involved in the study
☒	☐ Antibodies
☒	☐ Eukaryotic cell lines
☒	☐ Palaeontology and archaeology
☒	☐ Animals and other organisms
☒	☐ Clinical data
☒	☐ Dual use research of concern

Methods

n/a	Involved in the study
☒	☐ ChIP-seq
☒	☐ Flow cytometry
☒	☐ MRI-based neuroimaging
